# Supplementary material for: Correlation of Fabrication Methods and Enhanced Wear Performance in Nanoporous Anodic Aluminum Oxide with Incorporated Molybdenum Disulfide (MoS2) Nanomaterials
Source: Nanomaterials (Basel). 2024 Feb 29;14(5):451. doi: 10.3390/nano14050451 (PMC10934346; doi:10.3390/nano14050451)
Supplement: Supplementary file 1 [file nanomaterials-14-00451-s001.zip › nanomaterials-2863411-supplementary.pdf]

Supplementary Information for

Correlation of fabrication methods and enhanced wear performance in nanoporous anodic aluminum oxide with incorporated molybdenum disulfide (MoS_2) nanomaterials

Hatfield, Kendrich O'Donaghue[1]; Brown, Nathan[1]; Dervishi, Enkeleda[1]; Carpenter, Bradley[1]; Janusz, Jordyn[1]; Hooks, Daniel E. [1,2*]

1: SIGMA-2: Finishing Manufacturing Science, Los Alamos National Laboratory, SM-30 Bikini Atoll Road, Los Alamos, NM 87545, USA; khatfield@lanl.gov (K.O.H.)

2: MPA-CINT: Center for Integrated Nanotechnologies, Los Alamos National Laboratory, Los Alamos, NM 87545, USA

*Corresponding author, email: dhooks@lanl.gov, phone: 505-667-6407

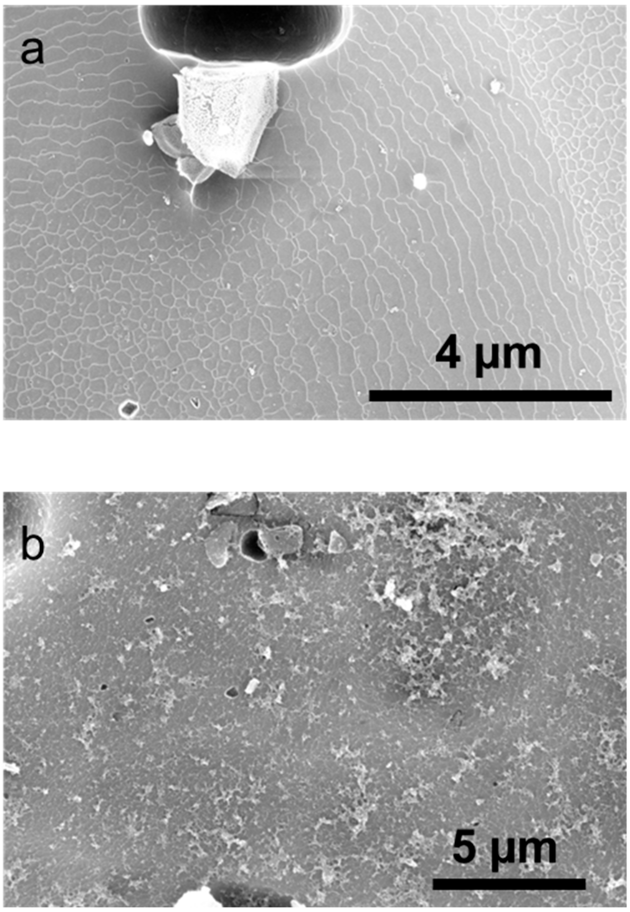

Figure S1. SEM images of 20-minute high-acid anodized a) unmodified AAO and b) MoS_2/AAO.

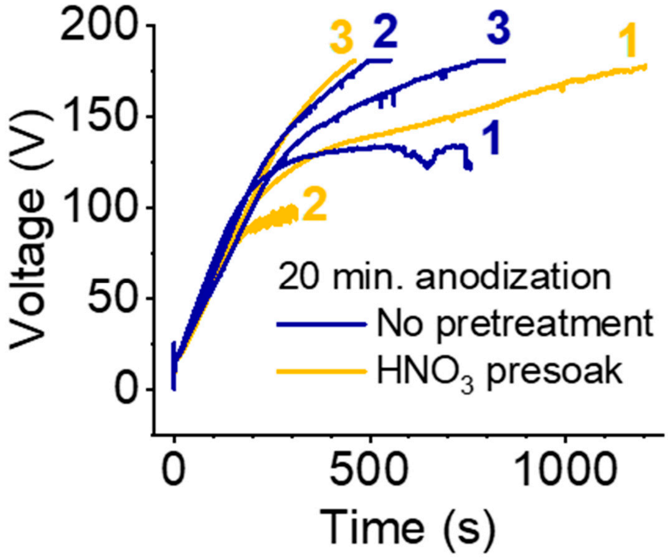

Figure S2. V-t deposition curves for 20-minute anodized Al 5052.

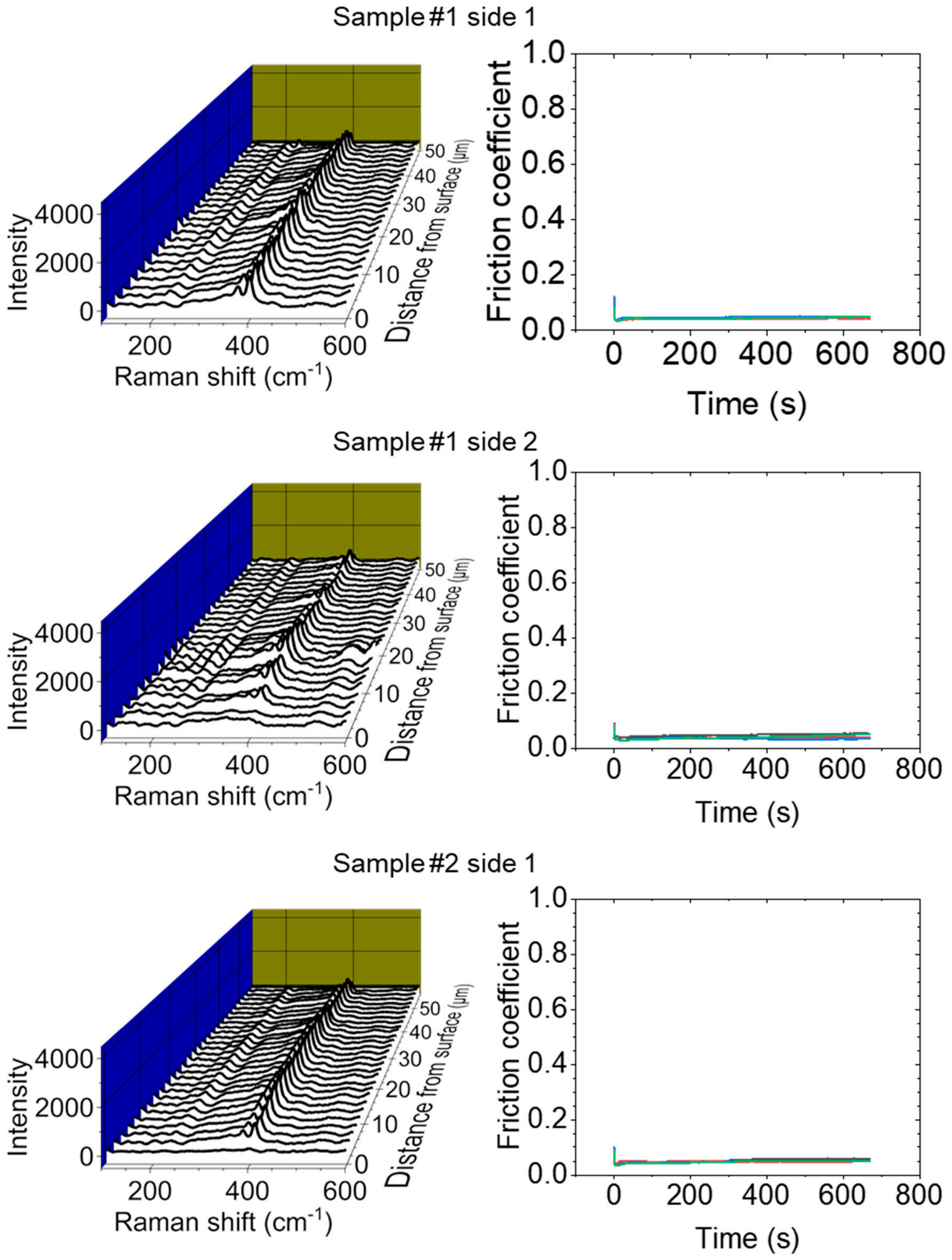

Figure S3. Cross-sectional Raman spectra and tribological scratch tests of 120-minute high-acid anodized Al 5052 samples with no acid pretreatment. Four scratches were performed per sample side.

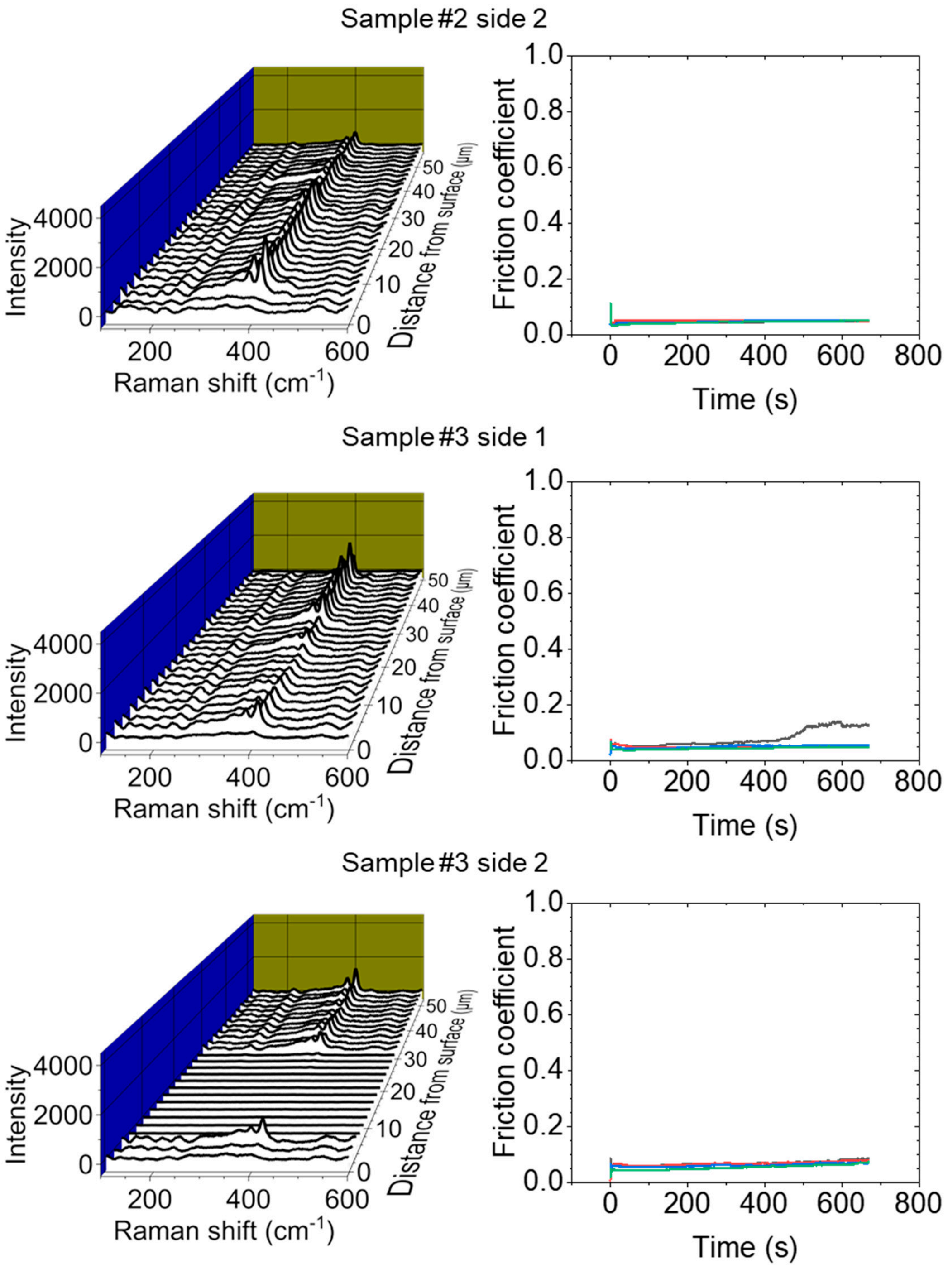

Figure S3 (continued). Cross-sectional Raman spectra and tribological scratch tests of 120-minute high-acid anodized Al 5052 samples with no acid pretreatment. Four scratches were performed per sample side.

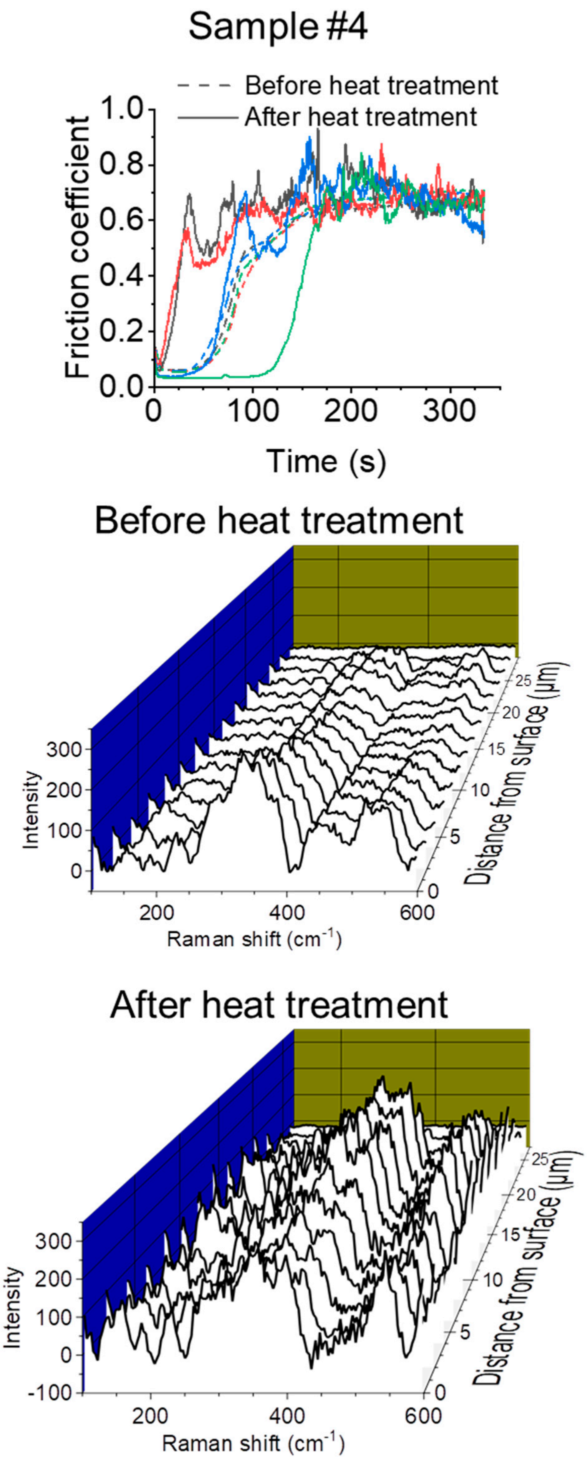

Figure S4. Cross-sectional Raman spectra and tribological scratch tests of 120-minute high-acid anodized Al 5052 samples with no acid pretreatment before and after heat treatment.

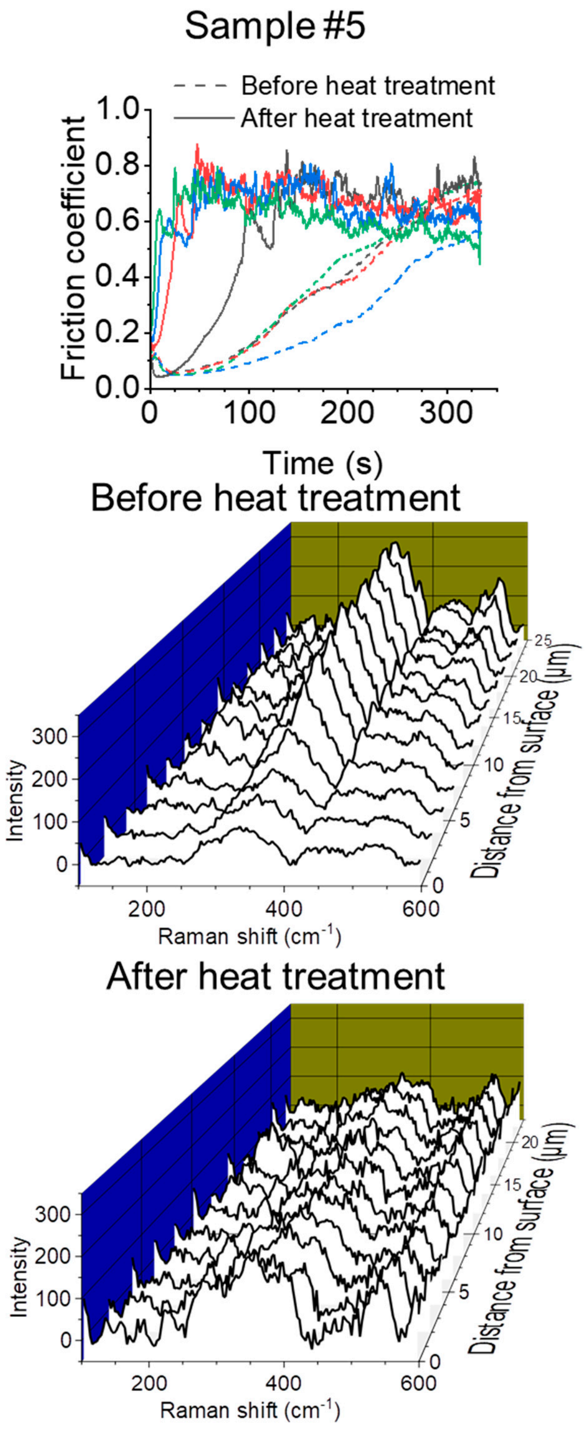

Figure S4 (continued). Cross-sectional Raman spectra and tribological scratch tests of 120-minute high-acid anodized Al 5052 samples with no acid pretreatment before and after heat treatment.

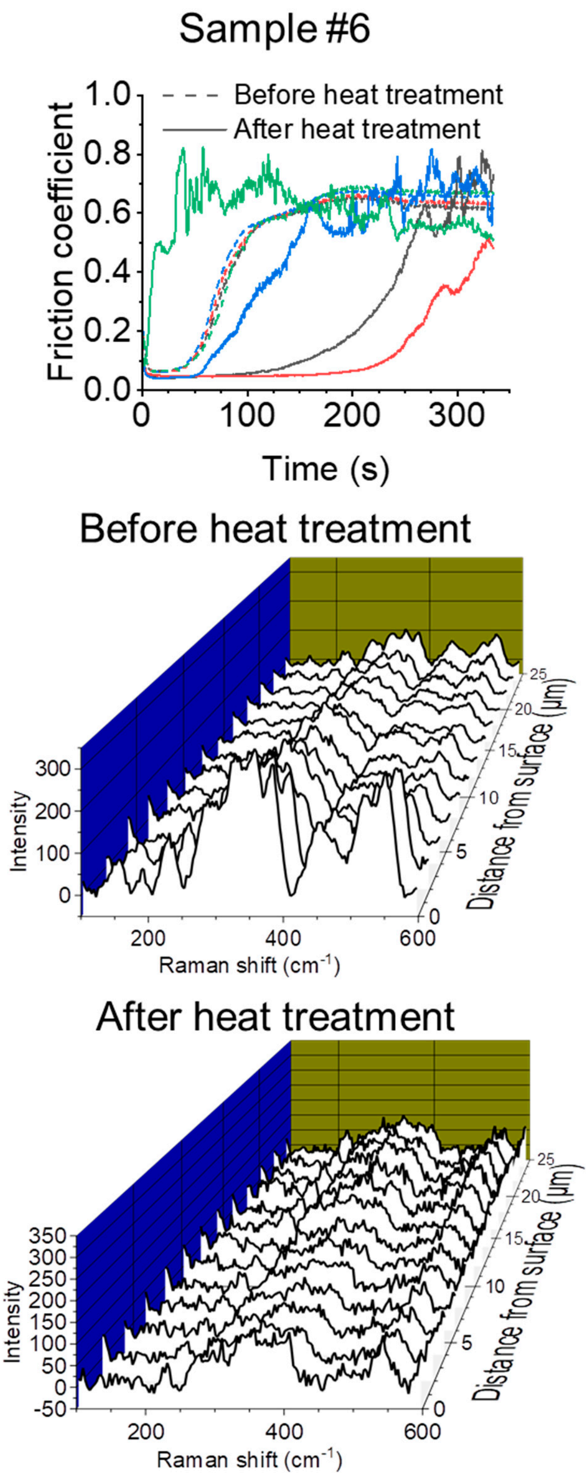

Figure S4 (continued). Cross-sectional Raman spectra and tribological scratch tests of 120-minute high-acid anodized Al 5052 samples with no acid pretreatment before and after heat treatment.

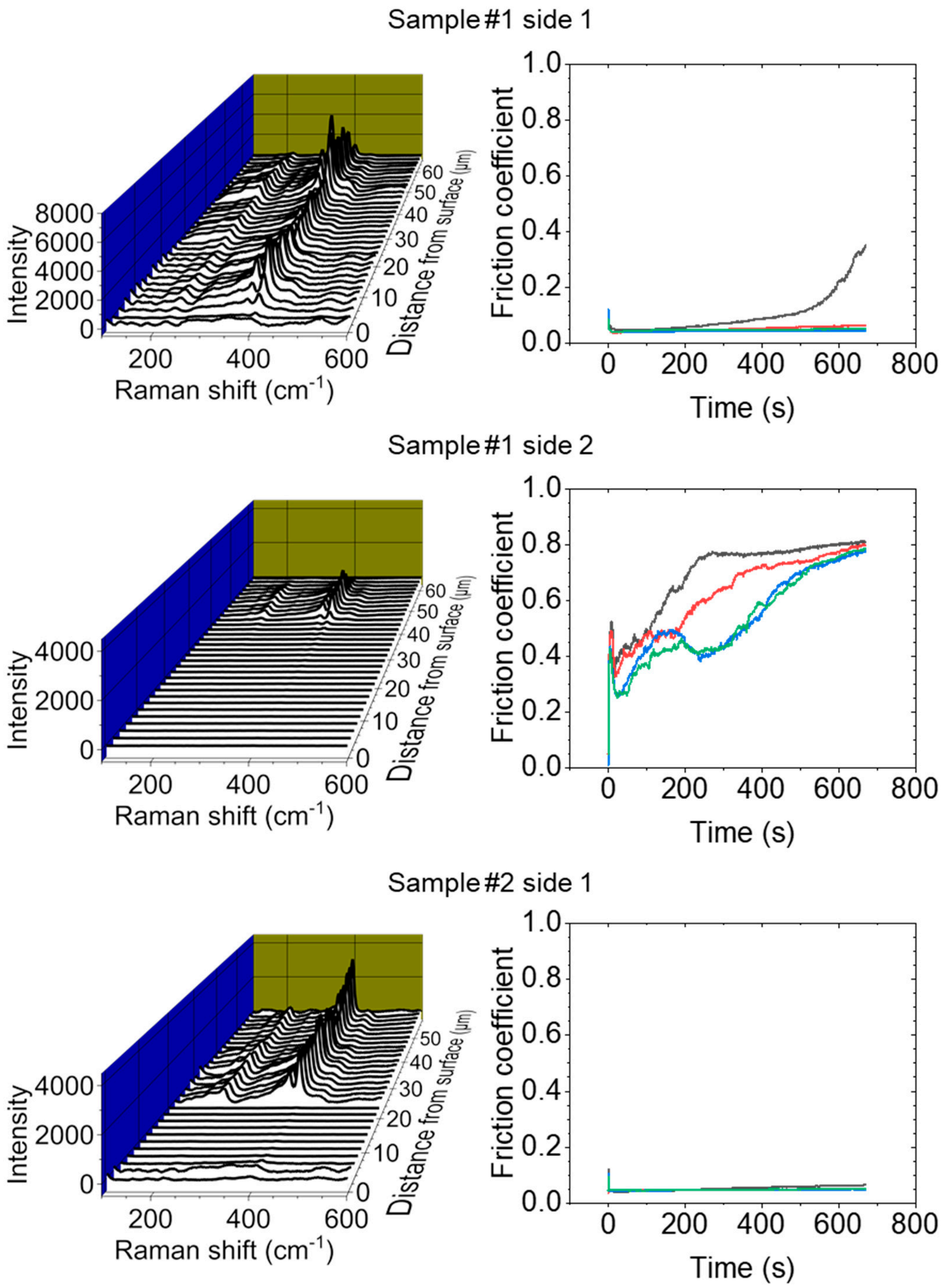

Figure S5. Cross-sectional Raman spectra and tribological scratch tests of 120-minute high-acid anodized Al 5052 samples with HNO_3 presoaking. Four scratches were performed per sample side.

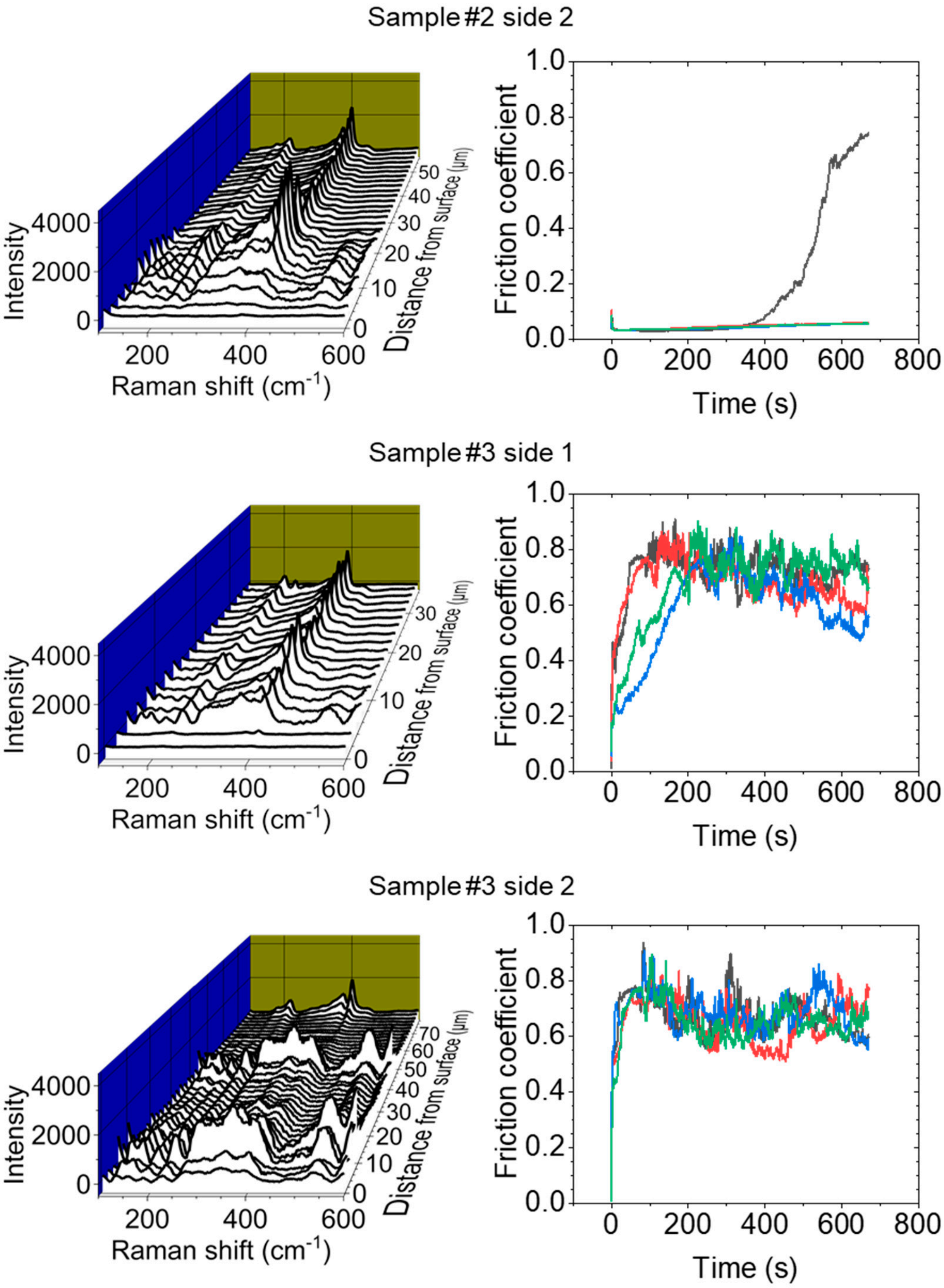

Figure S5 (continued). Cross-sectional Raman spectra and tribological scratch tests of 120-minute high-acid anodized Al 5052 samples with HNO_3 presoaking. Four scratches were performed per sample side.

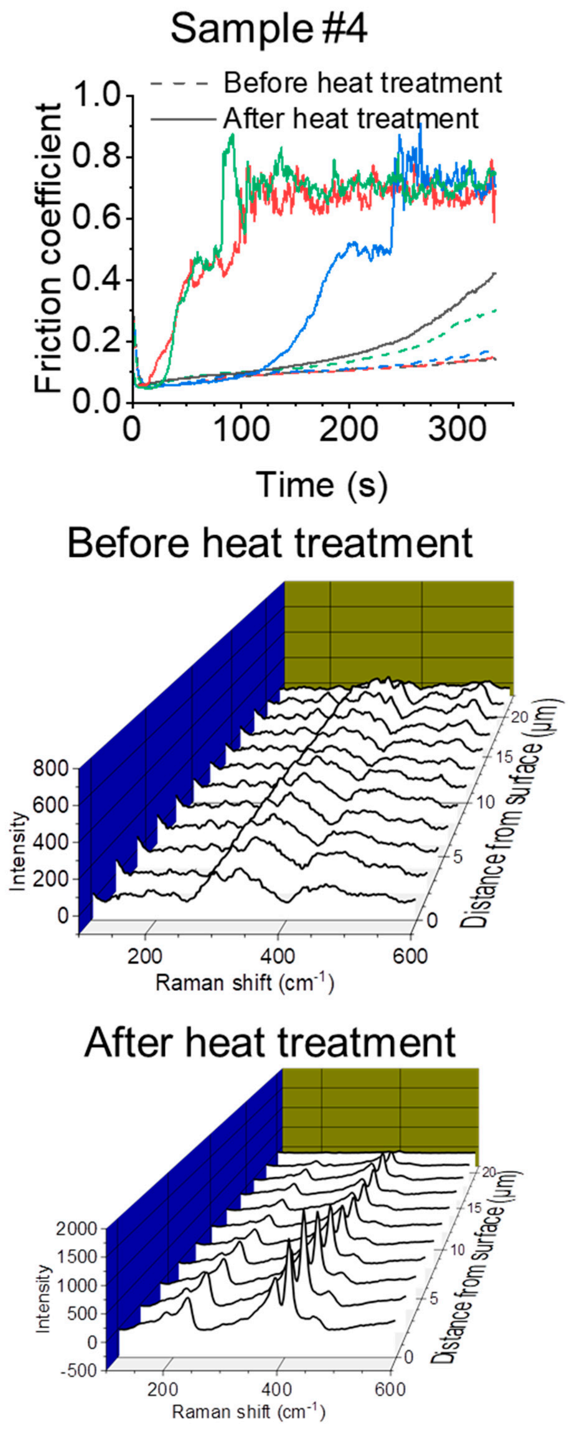

Figure S6. Cross-sectional Raman spectra and tribological scratch tests of 120-minute high-acid anodized Al 5052 samples with HNO_3 presoaking before and after heat treatment.

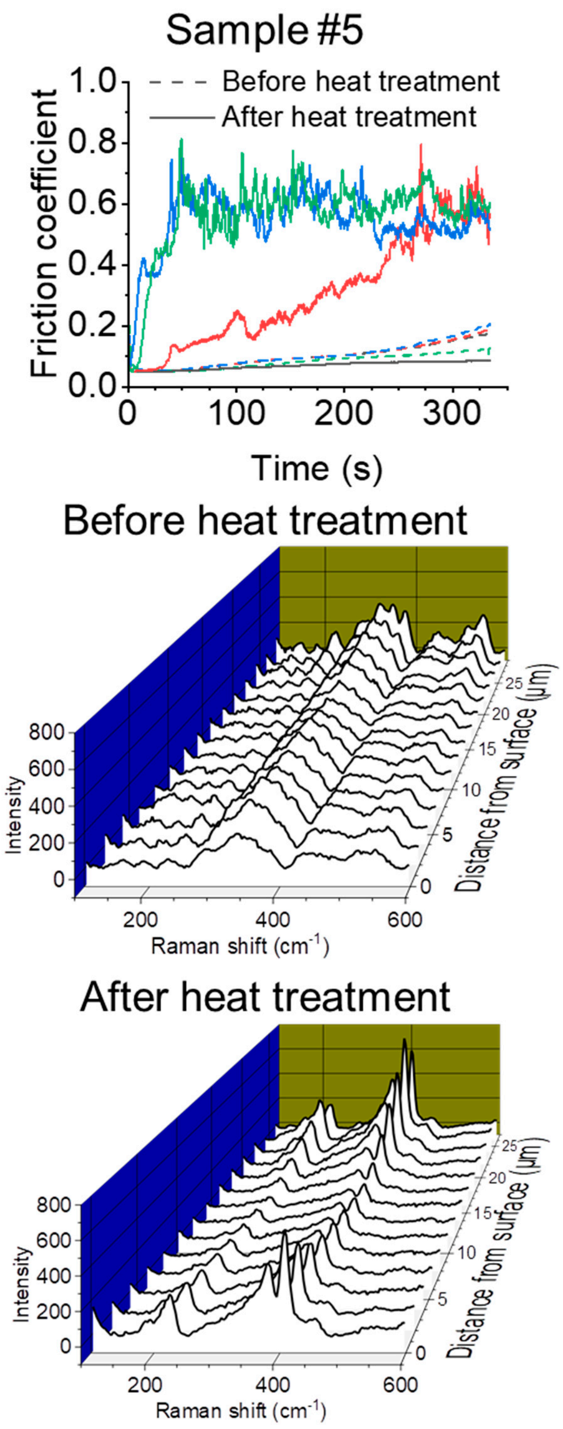

Figure S6 (continued). Cross-sectional Raman spectra and tribological scratch tests of 120-minute high-acid anodized Al 5052 samples with HNO_3 presoaking before and after heat treatment.

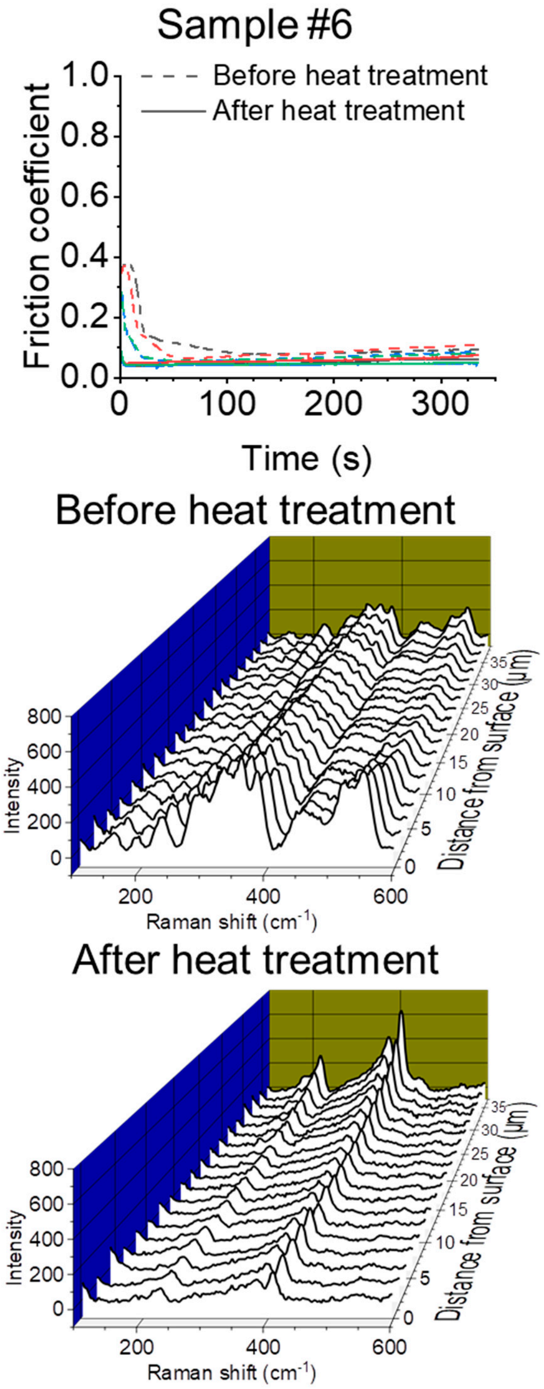

Figure S6 (continued). Cross-sectional Raman spectra and tribological scratch tests of 120-minute high-acid anodized Al 5052 samples with HNO_3 presoaking before and after heat treatment.

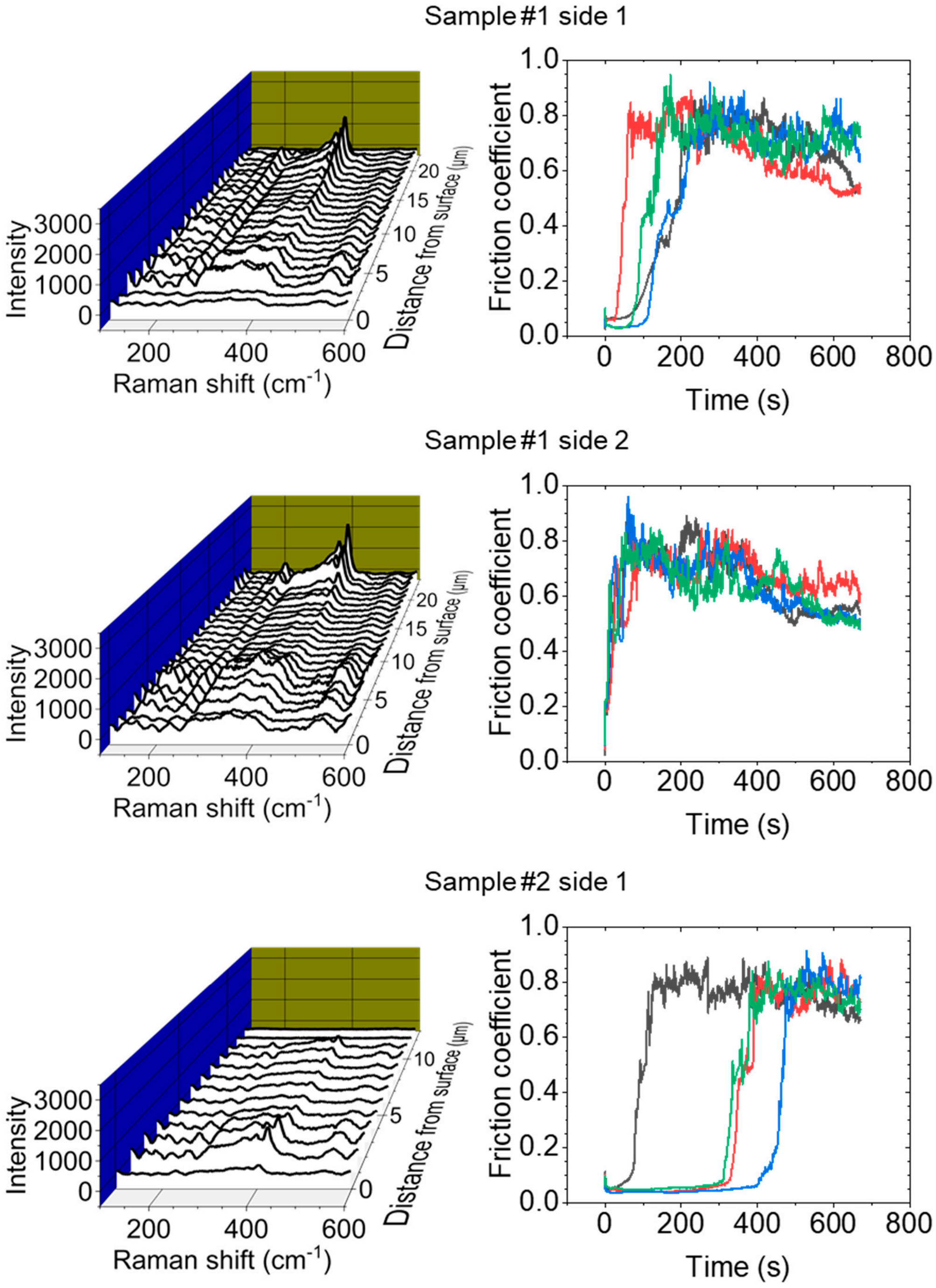

Figure S7. Cross-sectional Raman spectra and tribological scratch tests of 20-minute high-acid anodized Al 5052 samples with no acid pretreatment. Four scratches were performed per sample side.

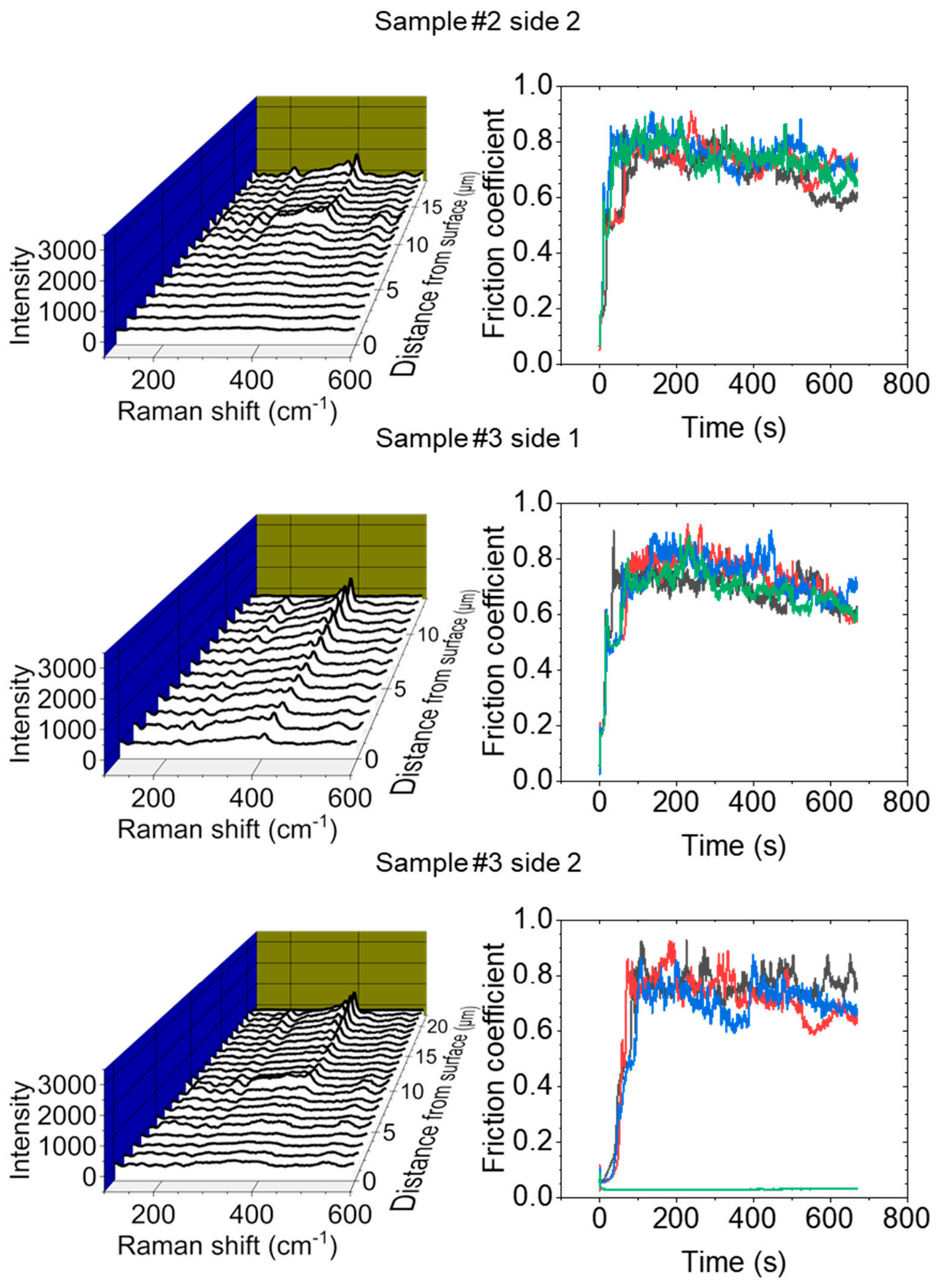

Figure S7. (continued): Cross-sectional Raman spectra and tribological scratch tests of 20-minute high-acid anodized Al 5052 samples with no acid pretreatment. Four scratches were performed per sample side.

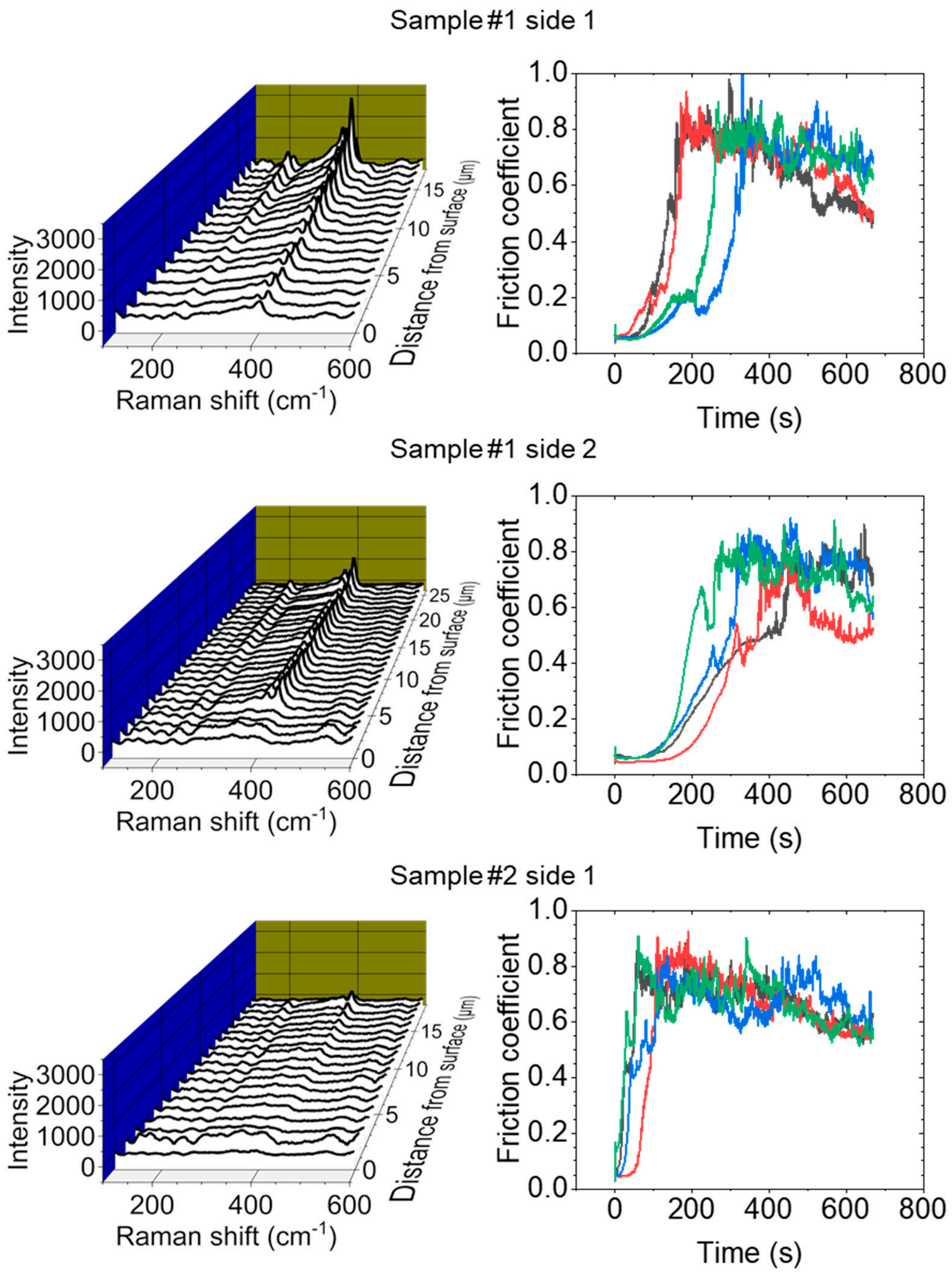

Figure S8. Cross-sectional Raman spectra and tribological scratch tests of 20-minute high-acid anodized Al 5052 samples with HNO_3 presoaking. Four scratches were performed per sample side.

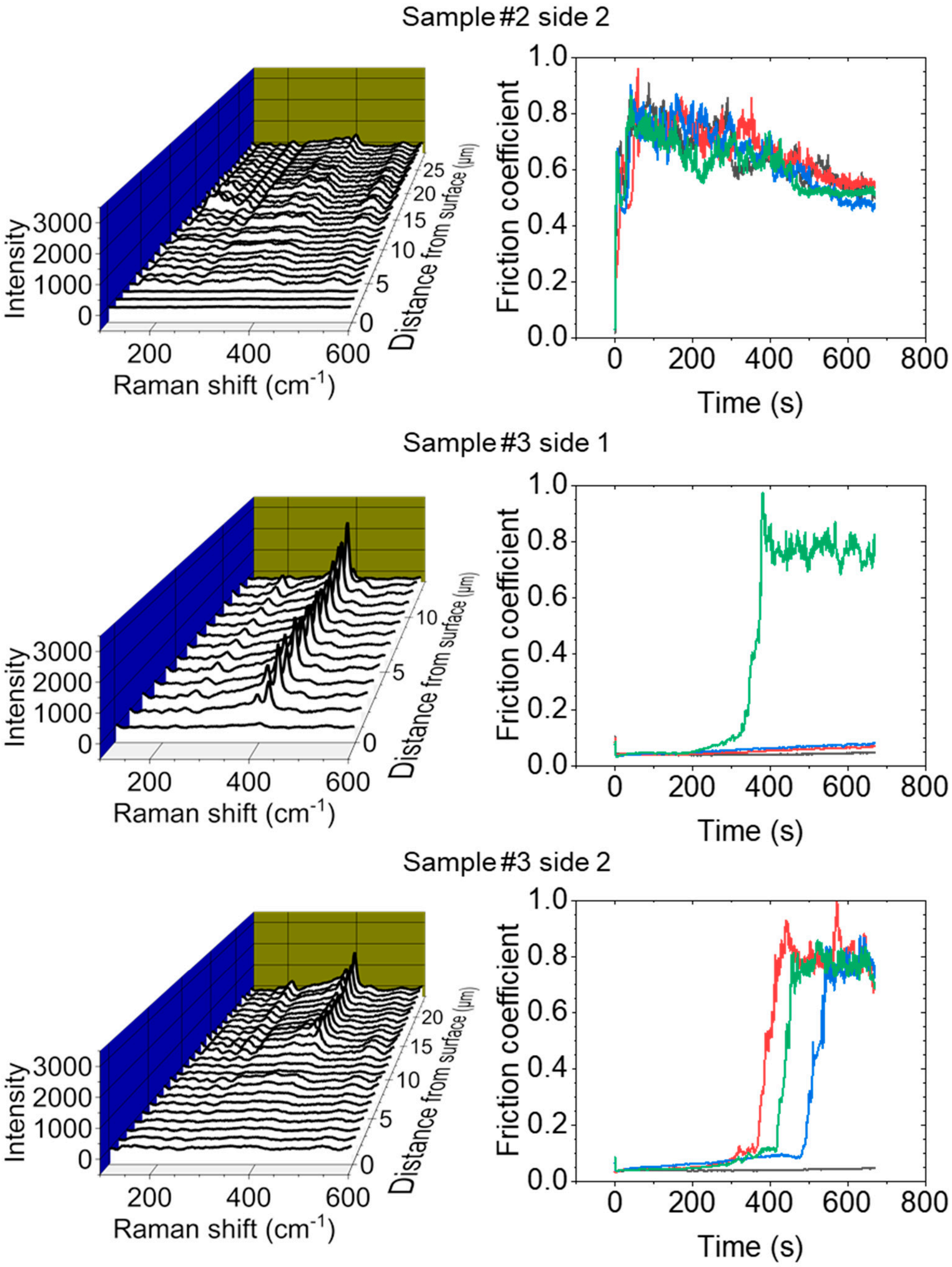

Figure S8. (continued): Cross-sectional Raman spectra and tribological scratch tests of 20-minute high-acid anodized Al 5052 samples with HNO3 presoaking. Four scratches were performed per sample side.

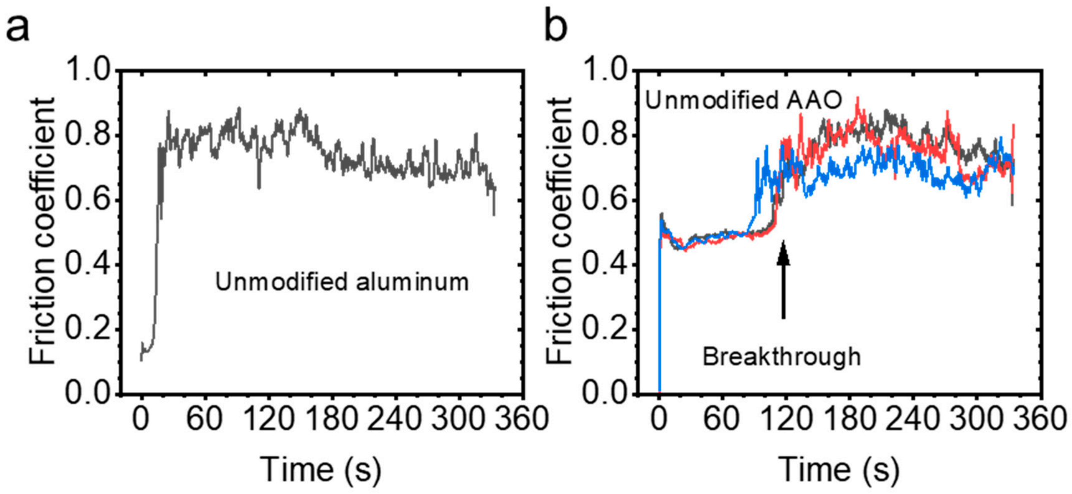

Figure S9. Reciprocating scratch tests of a) non-anodized Al 5052 and b) plain AAO from Al 5052.

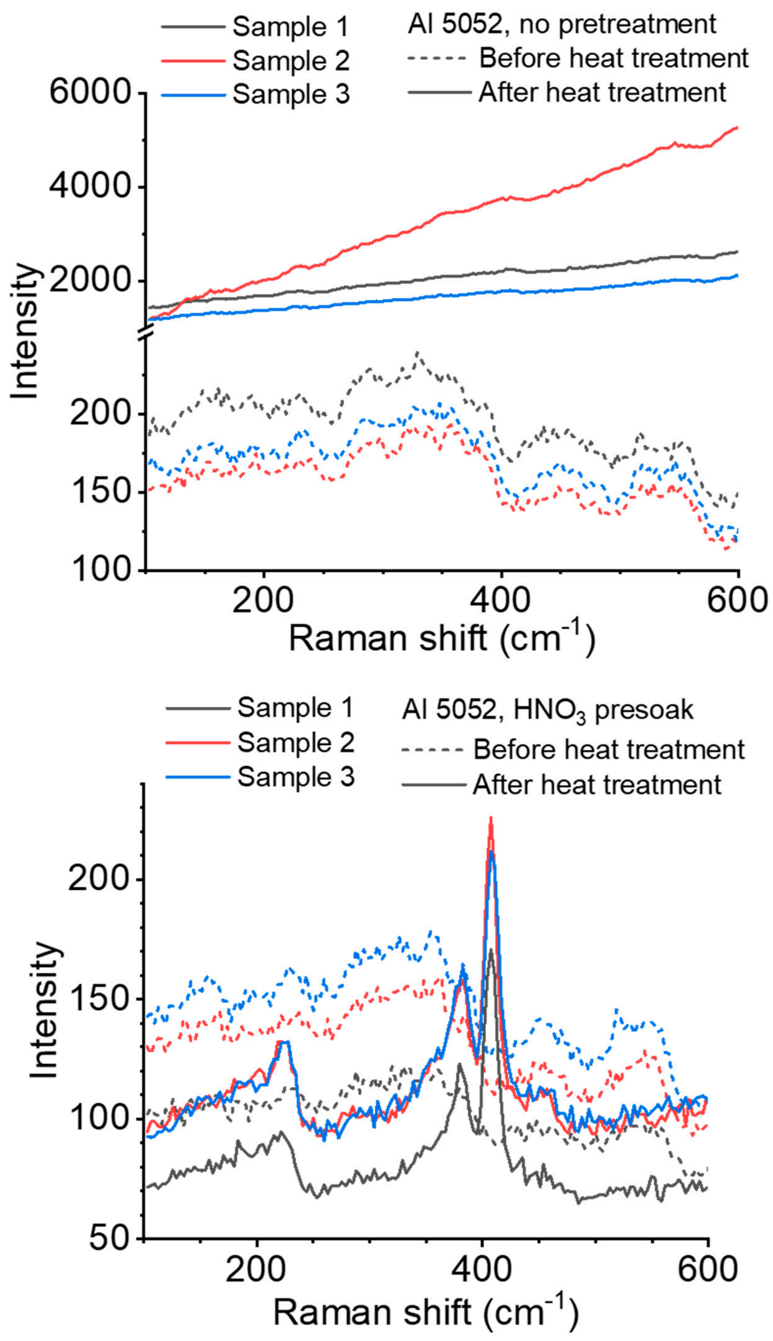

Figure S10. Raman spectra of Al 5052 MoS_2/AAO surfaces. No background subtraction was performed on these spectra.

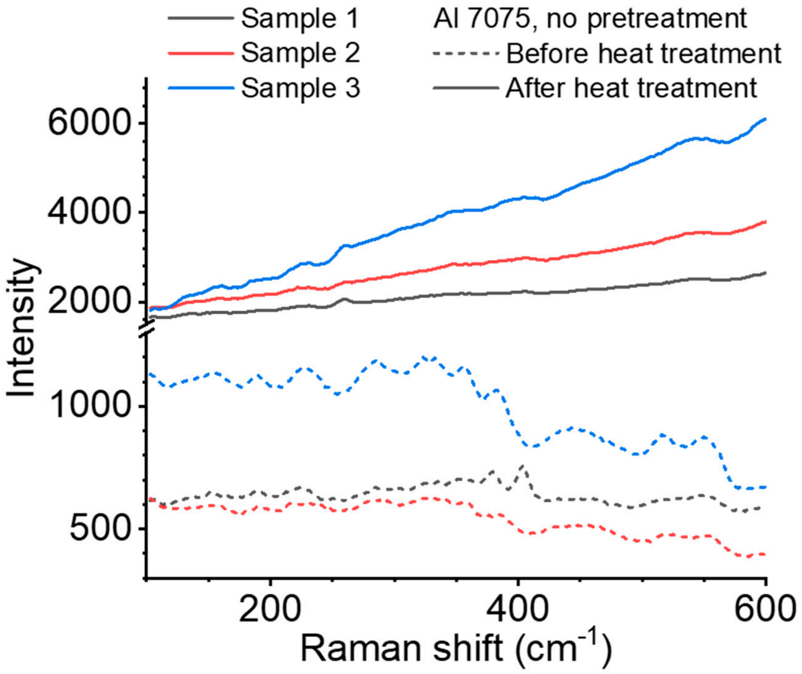

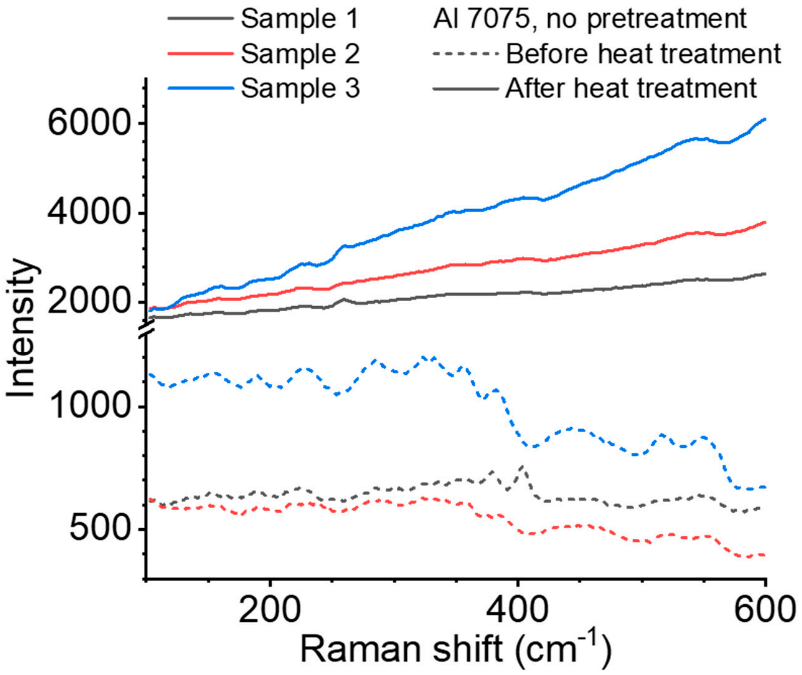

Figure S11. Raman spectra of Al 7075 MoS_2/AAO surfaces. No background subtraction was performed on these spectra.

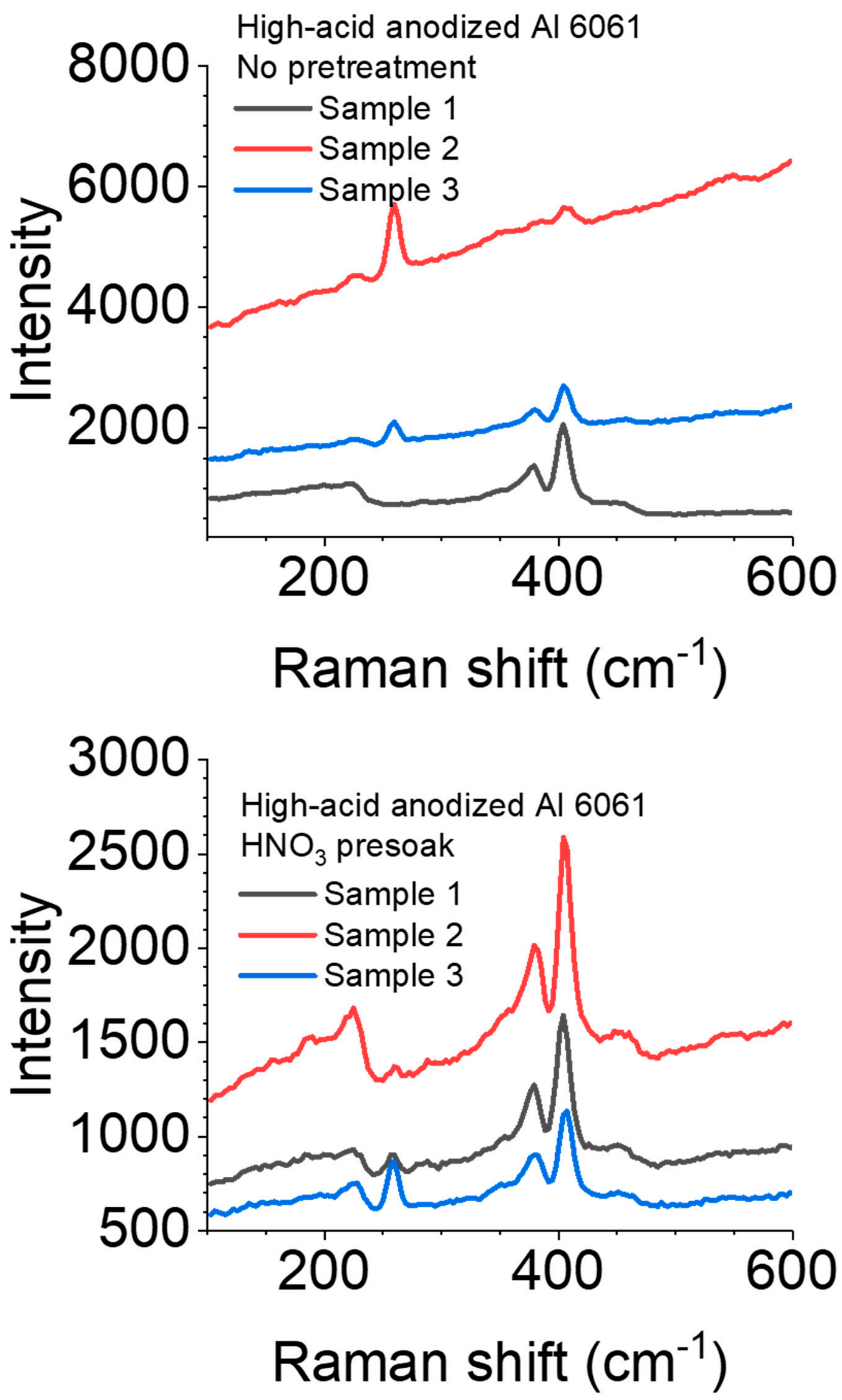

Figure S12. Raman spectra of Al 6061 MoS_2/AAO surfaces. No background subtraction was performed on these spectra.

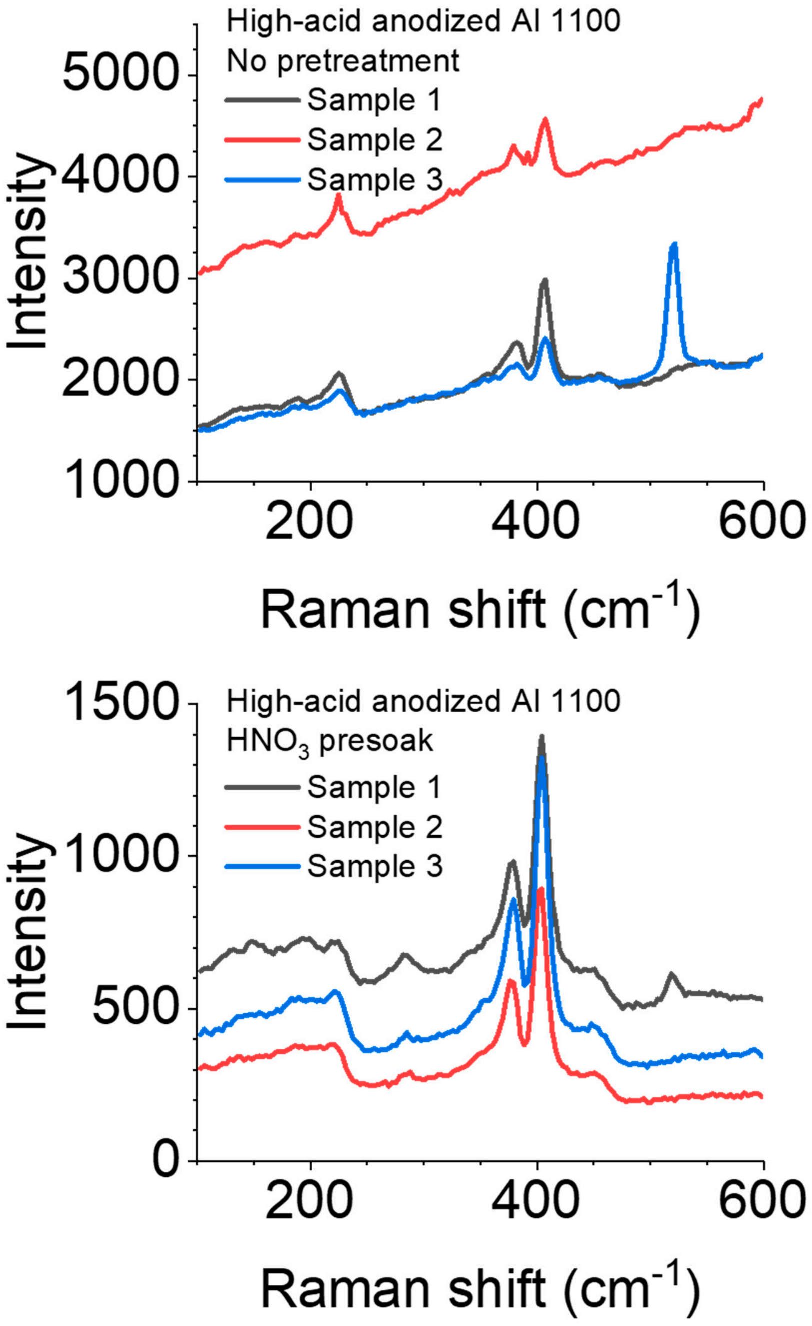

Figure S13. Raman spectra of Al 1100 MoS_2/AAO surfaces. No background subtraction was performed on these spectra.

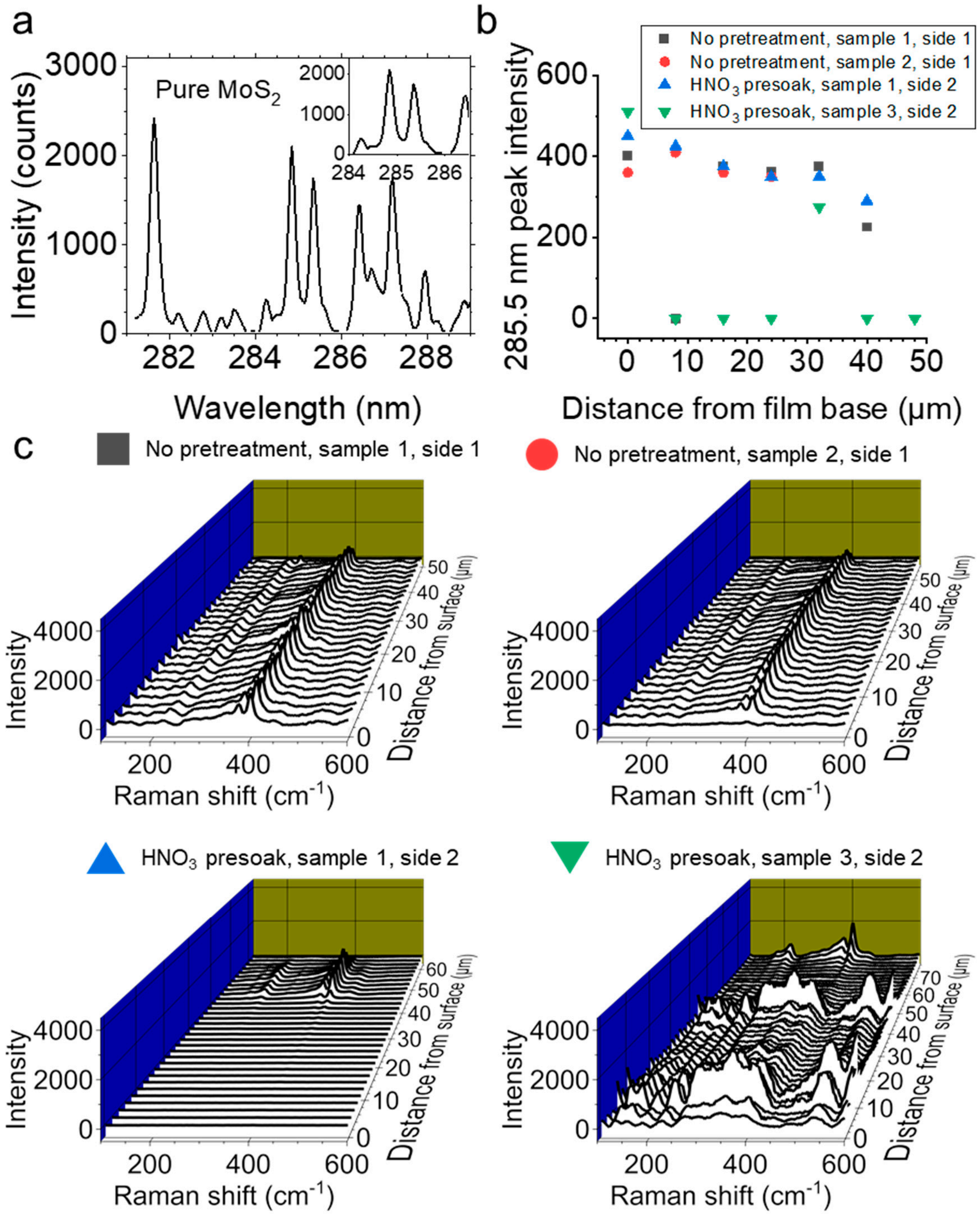

Figure S14. Laser-induced breakdown spectroscopy (LIBS) of MoS_2/AAO. a) LIBS spectrum of pure MoS_2 with inset showing a zoomed plot of the peaks near 285 nm. b) 285.5 nm peak intensity for various 120-minute high-acid anodized Al 5052 sample cross-sections. c) Cross-sectional Raman spectra for direct comparison between techniques.

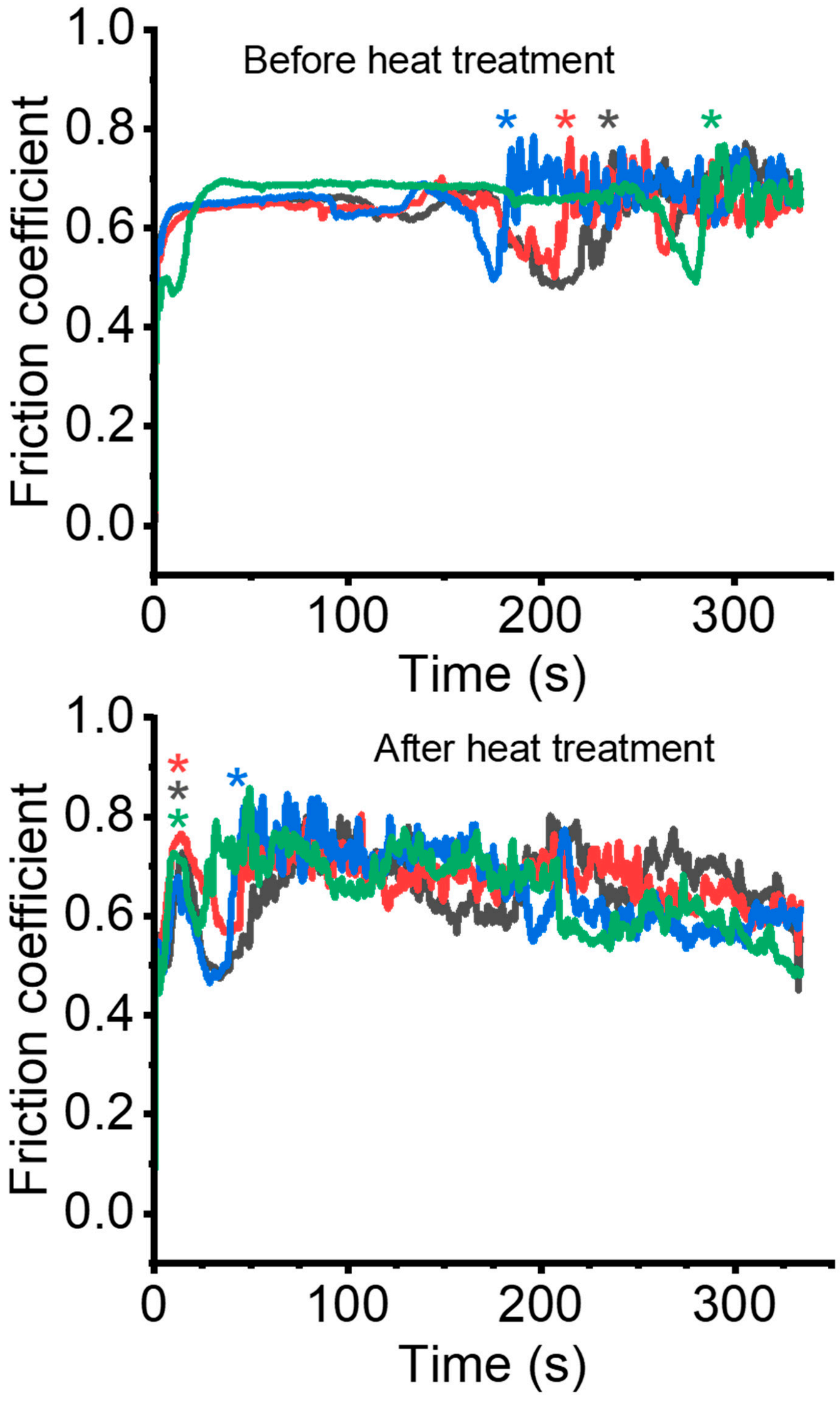

Figure S15. Reciprocating scratch tests of 120-minute high-acid anodized Al 5052 with no MoS_x deposition a) before and b) after heat treatment. Asterisks denote film breakthrough.

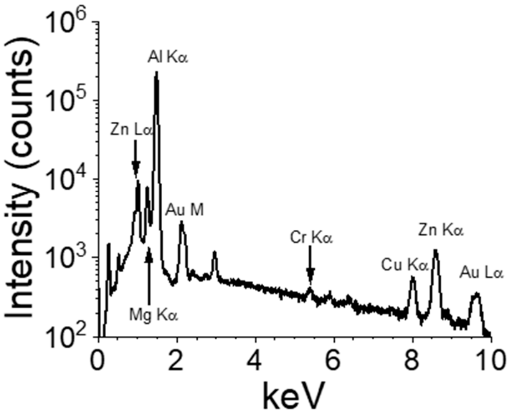

Element	Content (%)
Al	91.28 ± 0.73
Cr	0.39 ± 0.0.15
Fe	0.14 ± 0.06
Cu	1.75 ± 0.19
Zn	6.22 ± 0.55

Figure S16. Elemental analysis of the center of a commercial MoS_2/AAO part. a) EDS spectrum. b) X-ray fluorescence results.

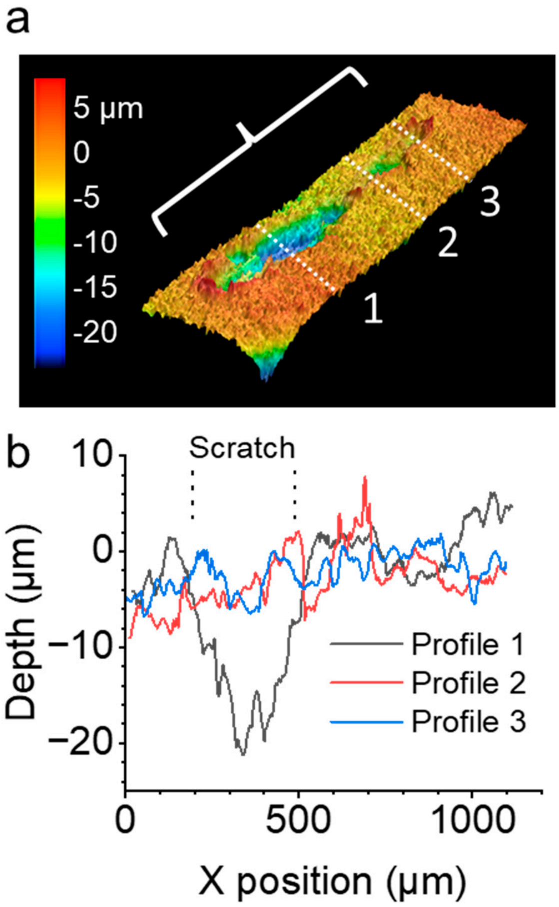

Figure S17. Example scratch of a commercial MoS_2/AAO film. a) 3-dimensional optical image of the scratch. b) Example depth profiles across the scratch in various areas.

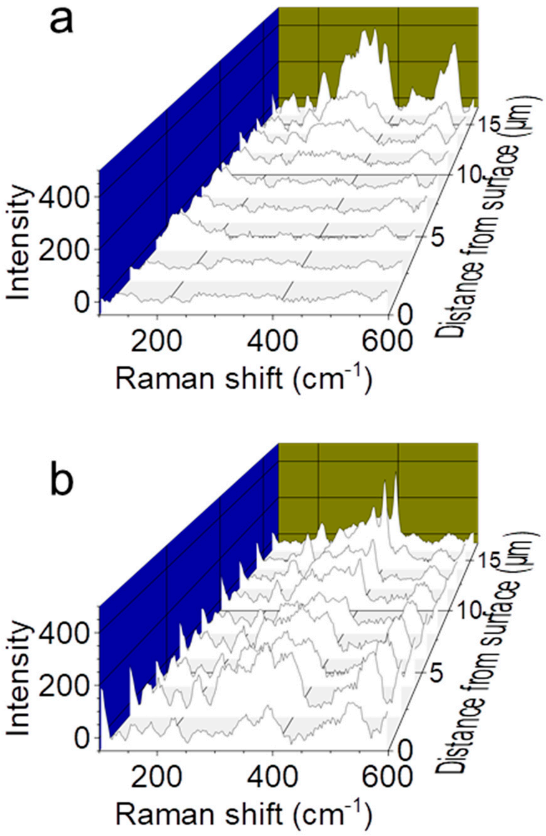

Figure S18. Raman linescans of a commercial MoS_2/AAO film before (a) and after (b) heat treatment.

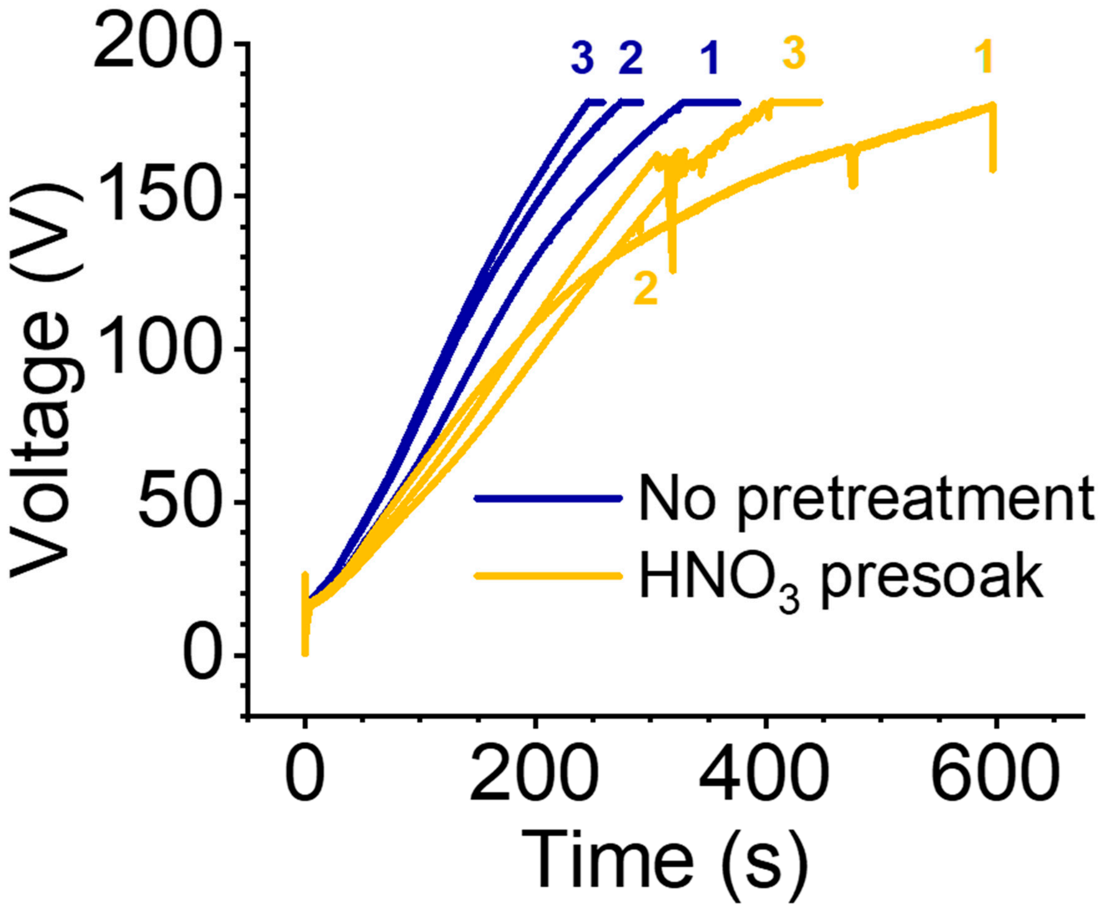

Figure S19. V-t curves for MoS_x deposition on high-acid 120-minute anodized Al 7075, run in triplicate (sample numbers denoted on the graph).

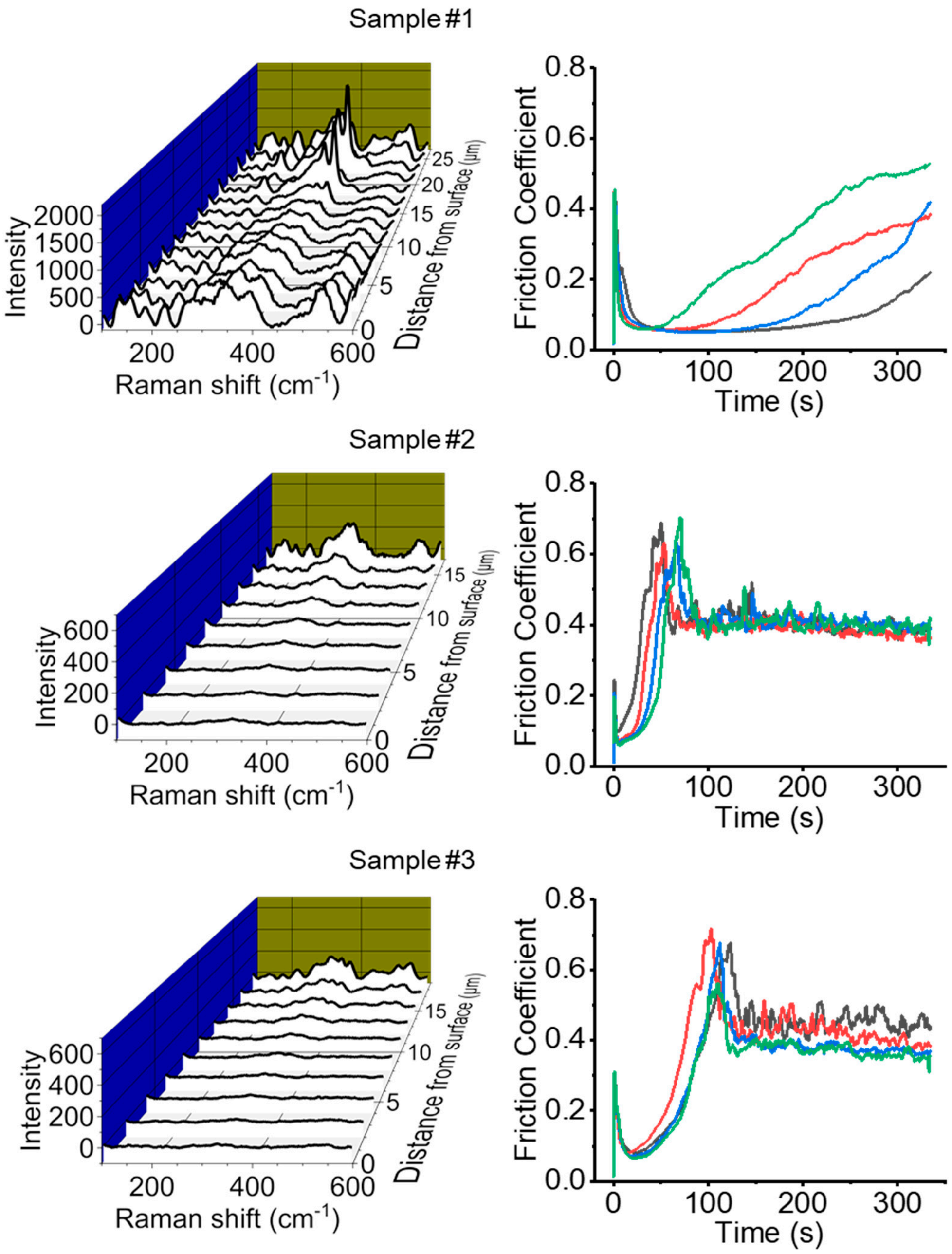

Figure S20. Raman-tribology comparison of 120-minute high-acid anodized Al 7075 samples with no acid pretreatment before heat treatment. Four scratches were performed per sample.

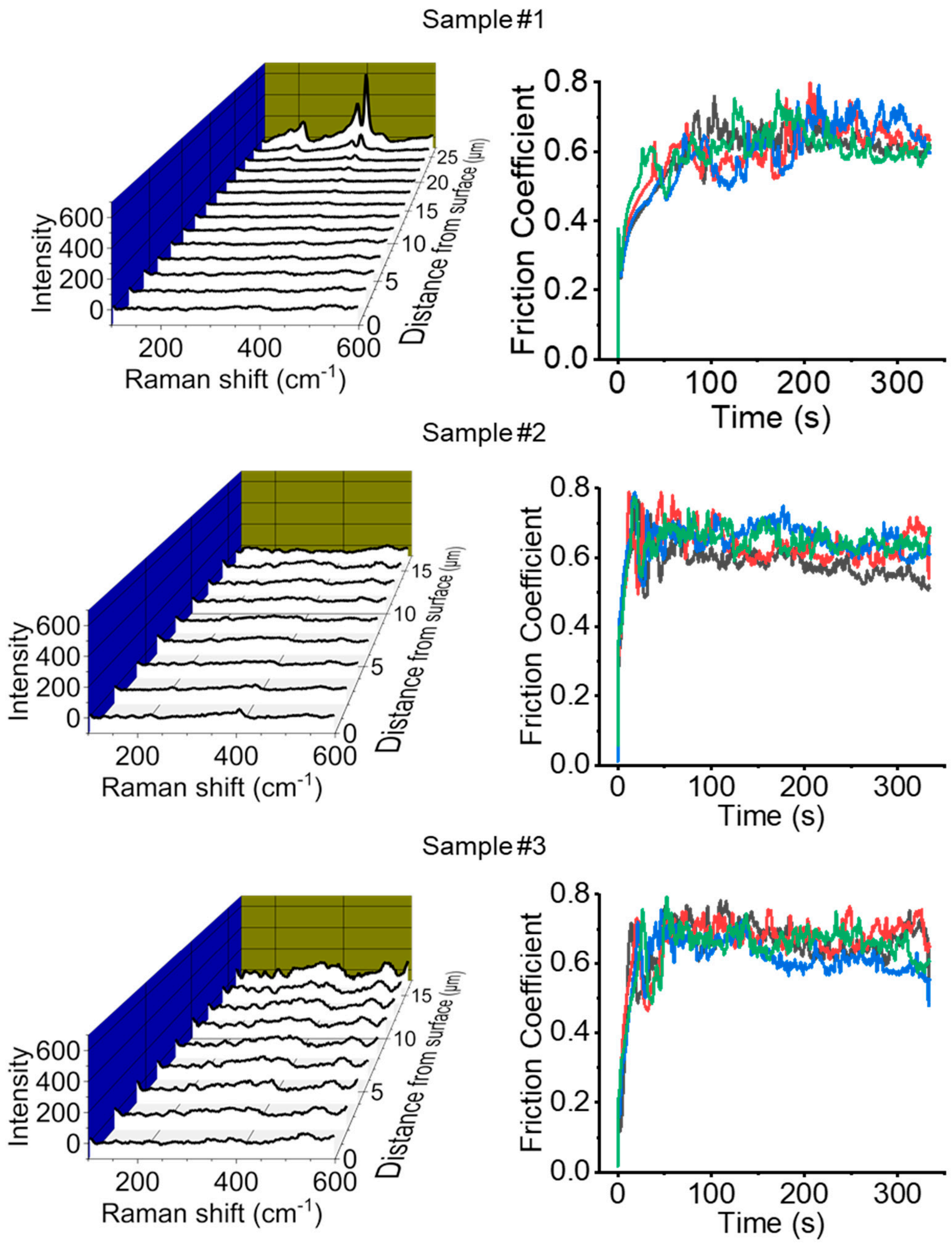

Figure S21. Raman-tribology comparison of 120-minute high-acid anodized Al 7075 samples with no acid pretreatment after heat treatment. Four scratches were performed per sample.

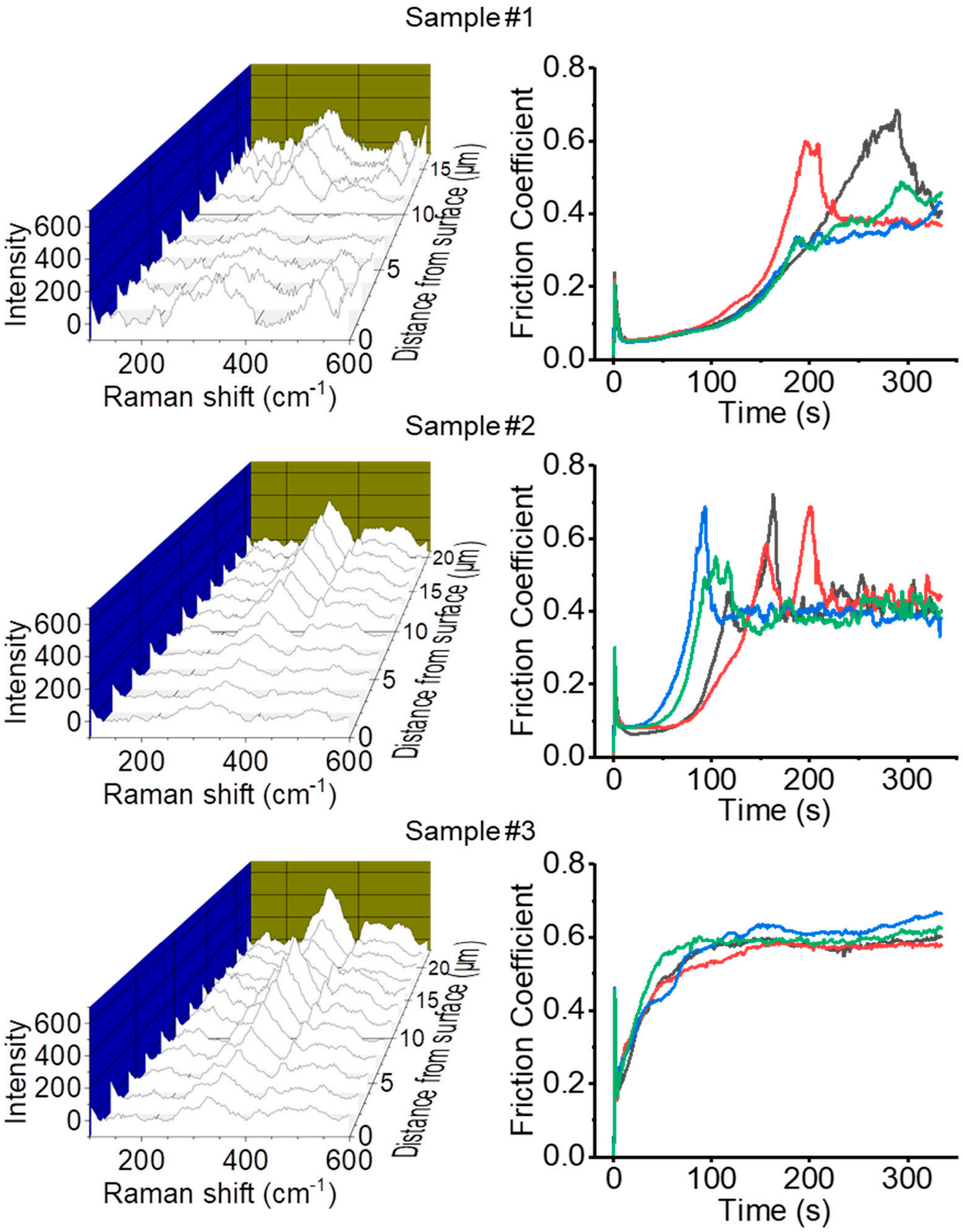

Figure S22. Raman-tribology comparison of 120-minute high-acid anodized Al 7075 samples with HNO_3 presoaking before heat treatment. Four scratches were performed per sample.

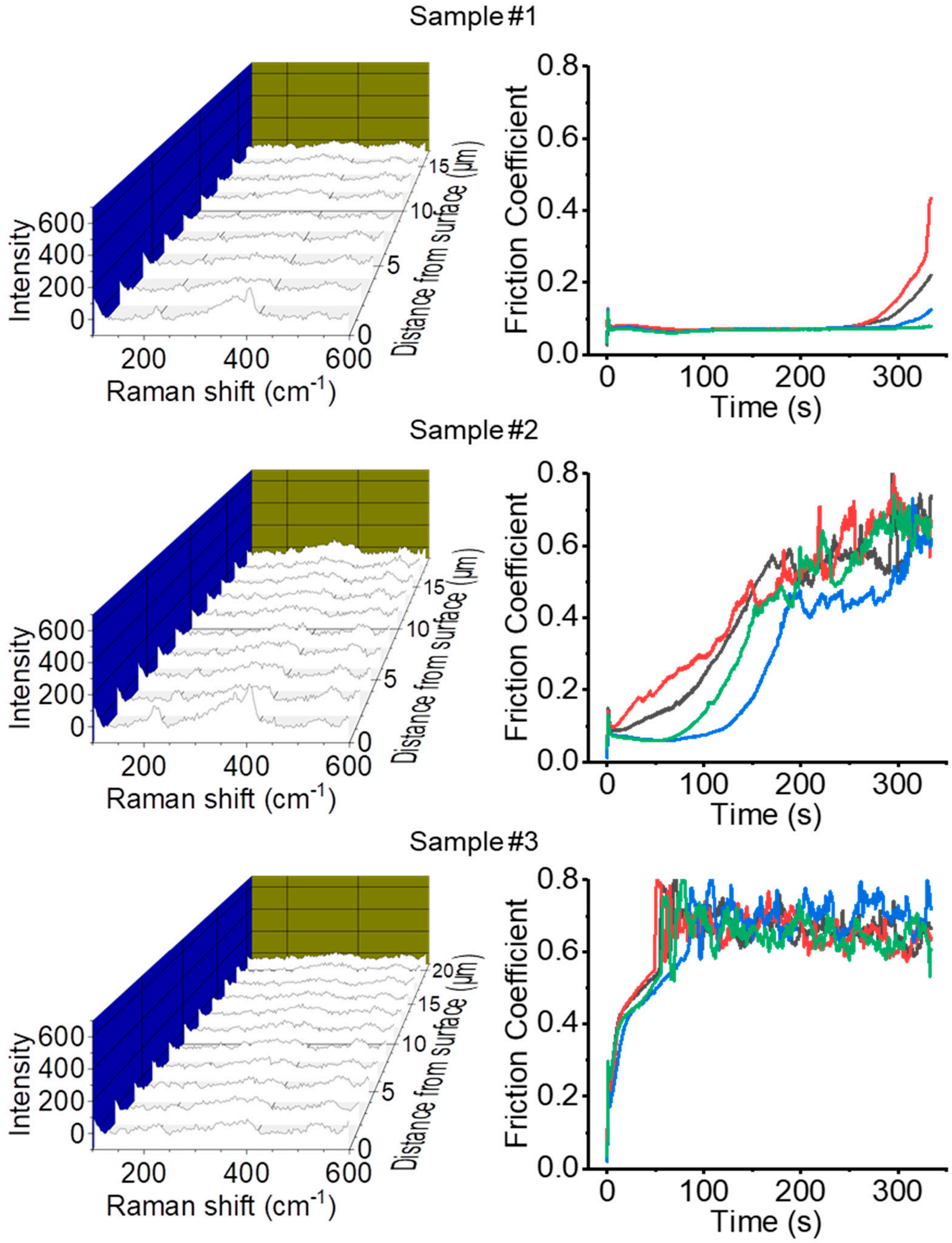

Figure S23. Raman-tribology comparison of 120-minute high-acid anodized Al 7075 samples with HNO_3 presoaking <u>after heat treatment</u>. Four scratches were performed per sample.

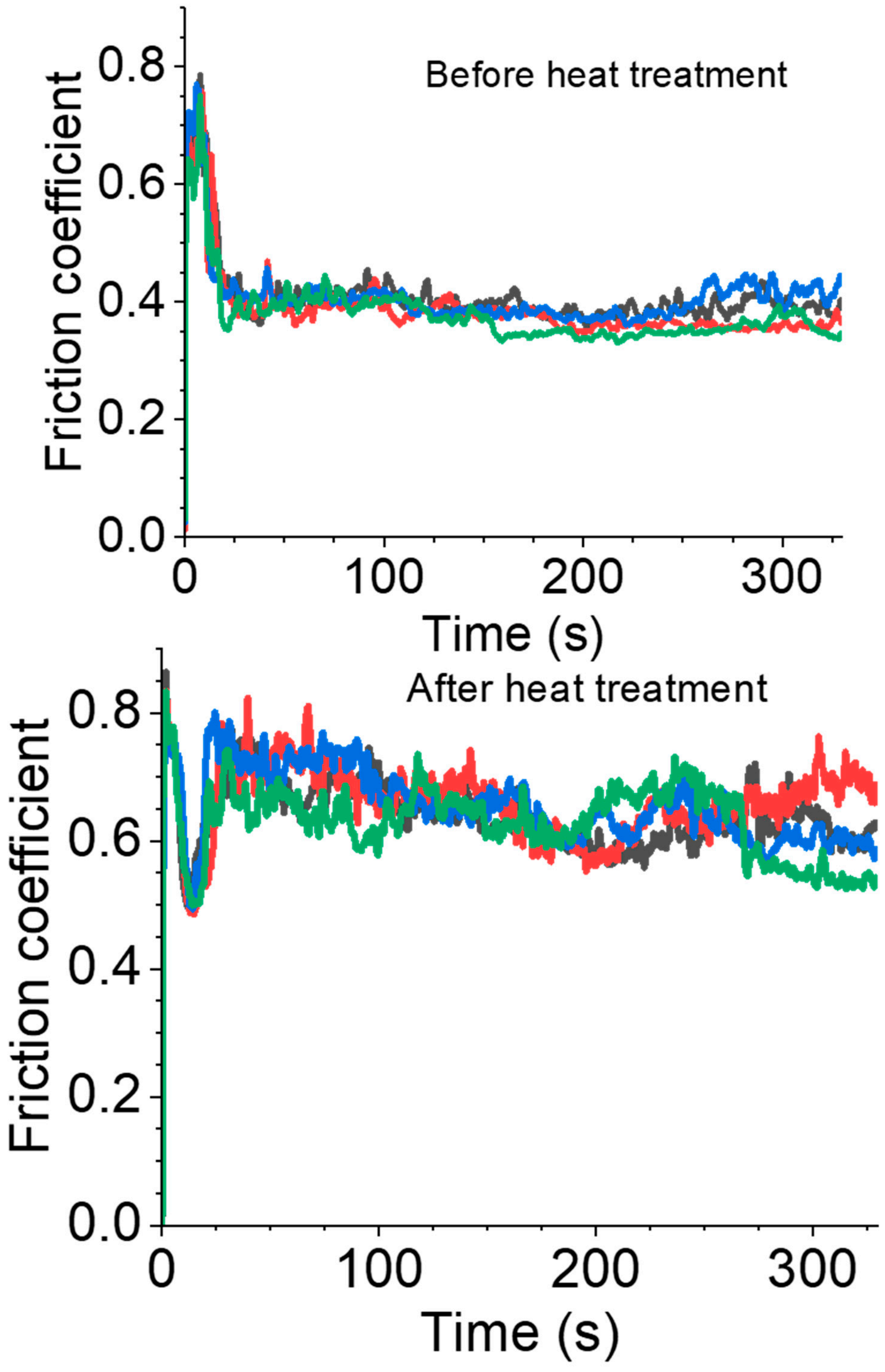

Figure S24. Reciprocating scratch tests of 120-minute high-acid anodized Al 7075 with no MoS_x deposition a) before and b) after heat treatment. Asterisks denote film breakthrough.

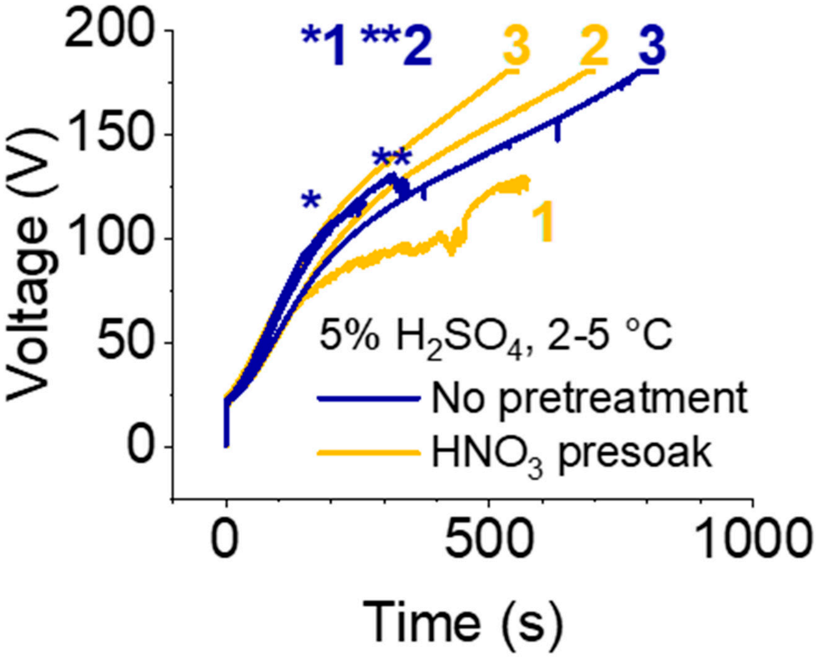

Figure S25. V-t curves for MoS_x deposition on low-acid anodized Al 5052 (5% H_2SO_4 at 2-5 °C) for 120 minutes. All samples were run in triplicate (sample numbers denoted in the figure).

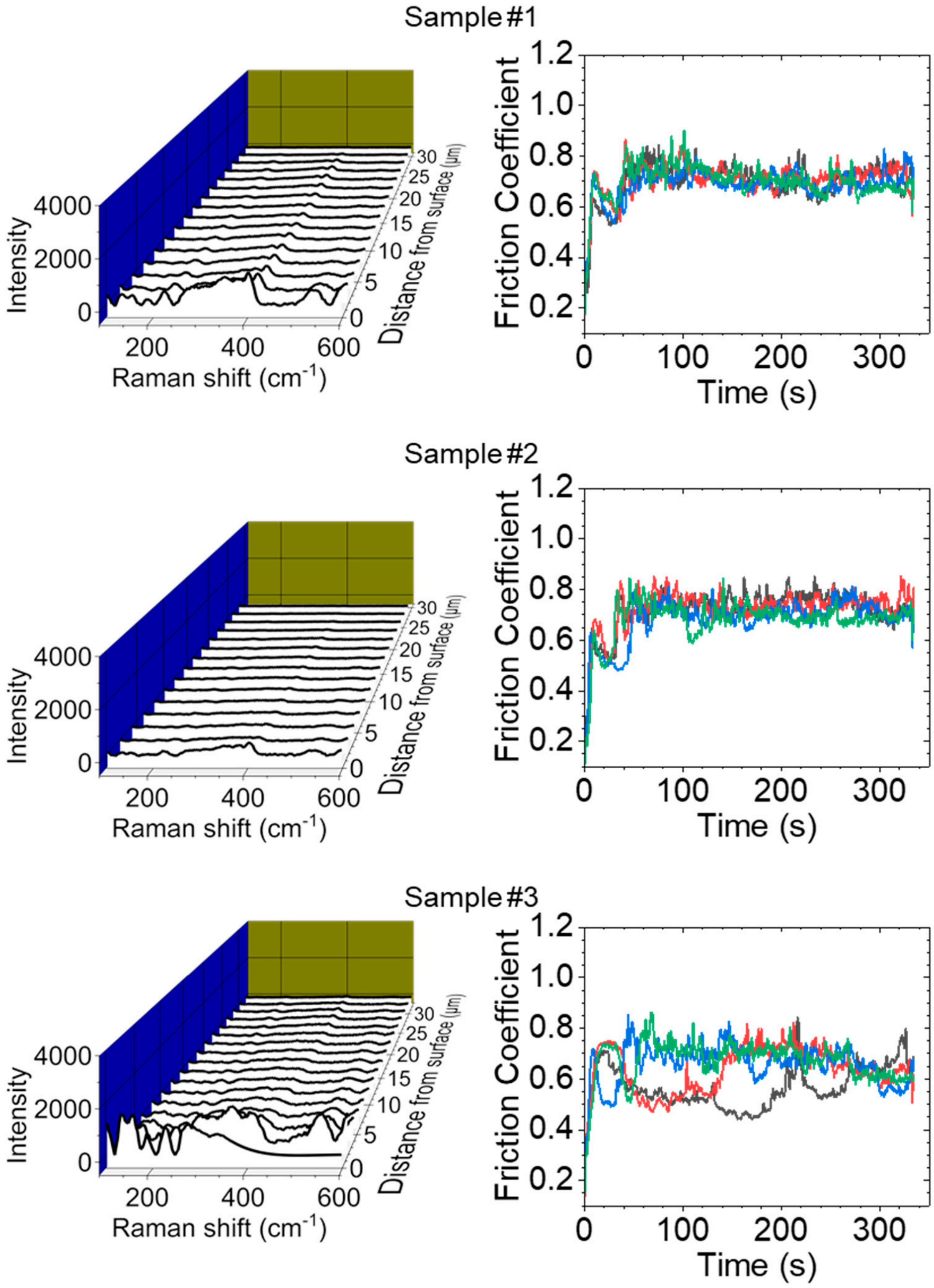

Figure S26. Raman-tribology comparison of 120-minute low-acid anodized Al 5052 samples with no acid pretreatment. Four scratches were performed per sample.

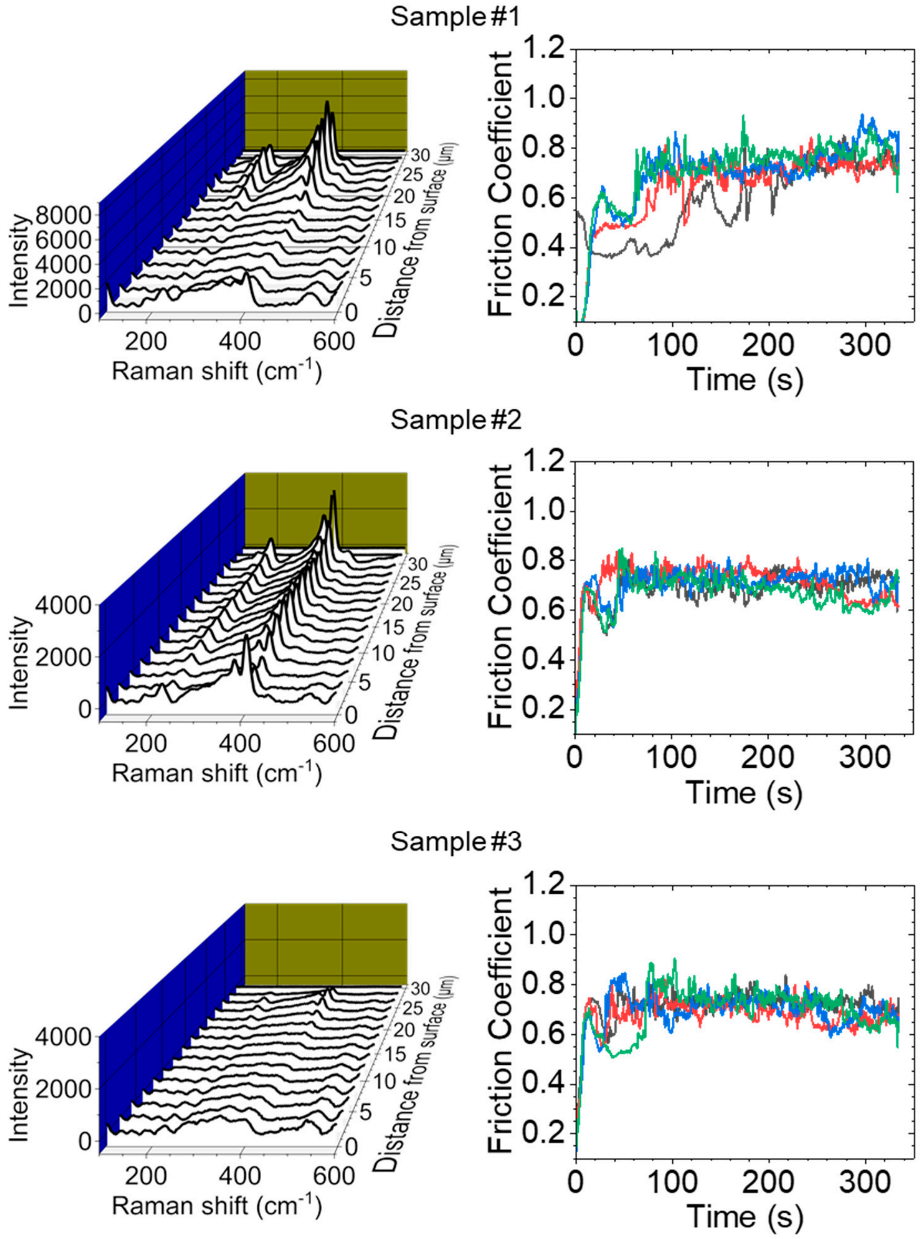

Figure S27. Raman-tribology comparison of 120-minute low-acid anodized Al 5052 samples with HNO_3 pretreatment. Four scratches were performed per sample.

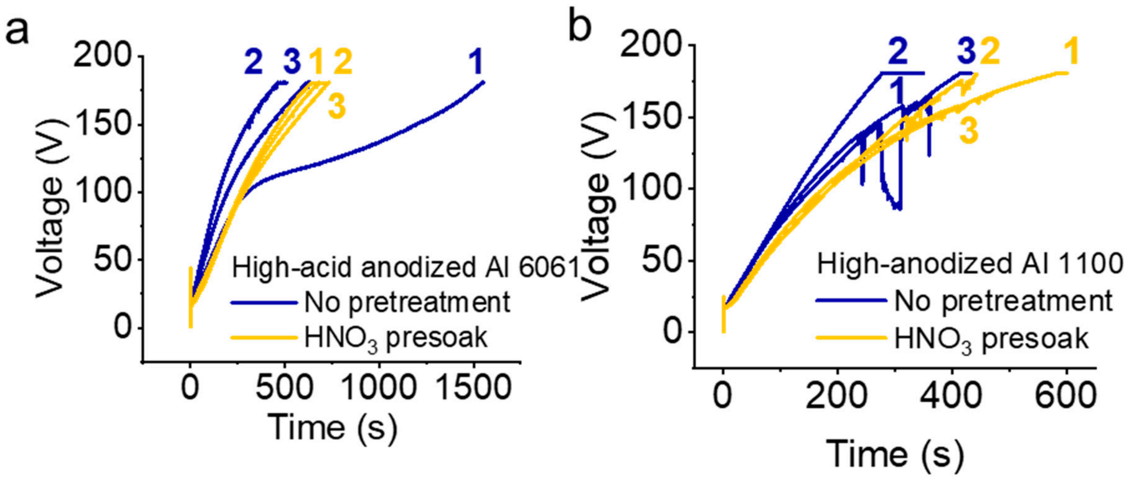

Figure S28. V-t curves for MoS_x deposition on a) Al 6061 and b) Al 1100. All samples were run in triplicate (sample numbers denoted in the figure).

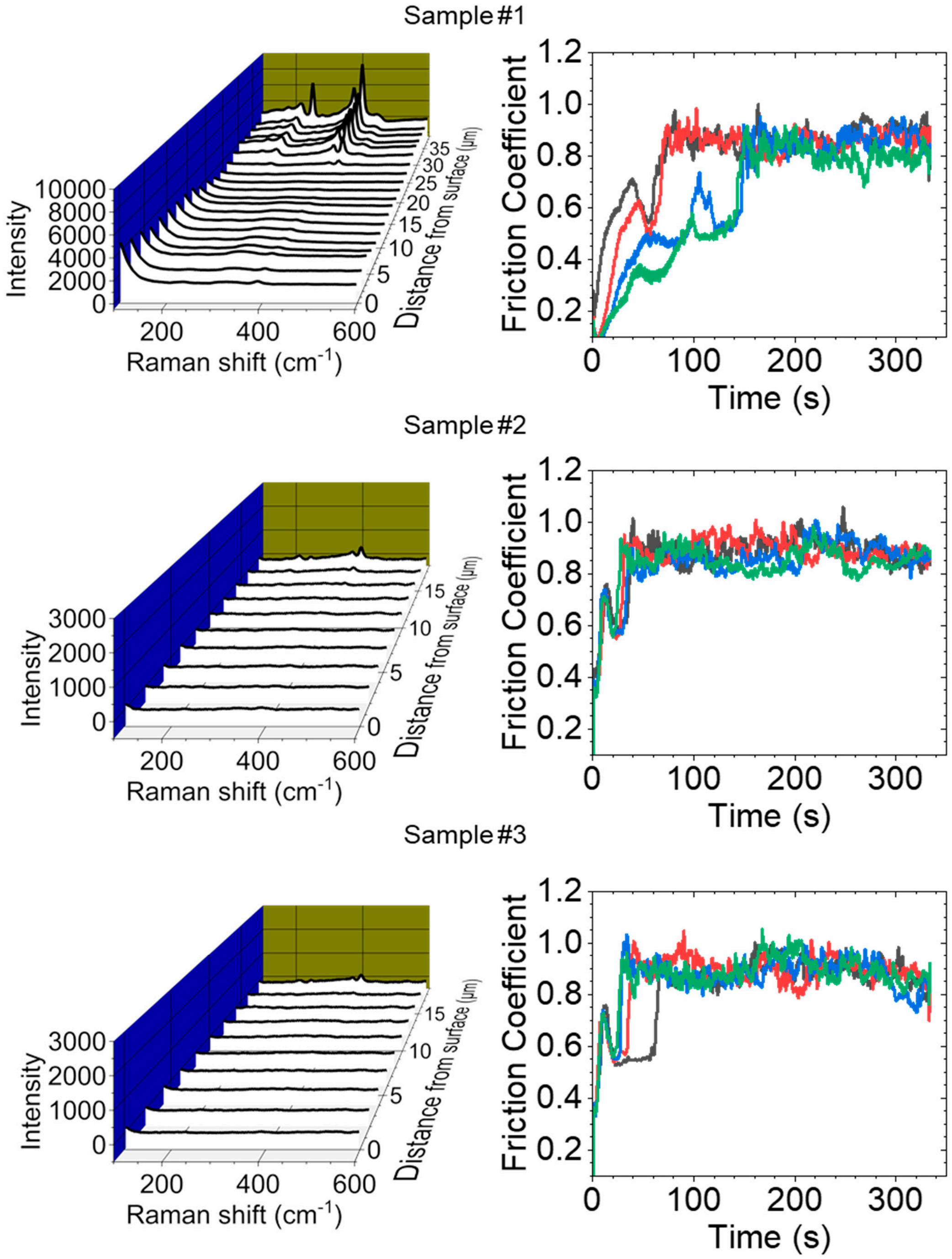

Figure S29. Raman-tribology comparison of 120-minute high-acid anodized Al 6061 samples with no acid pretreatment. Four scratches were performed per sample.

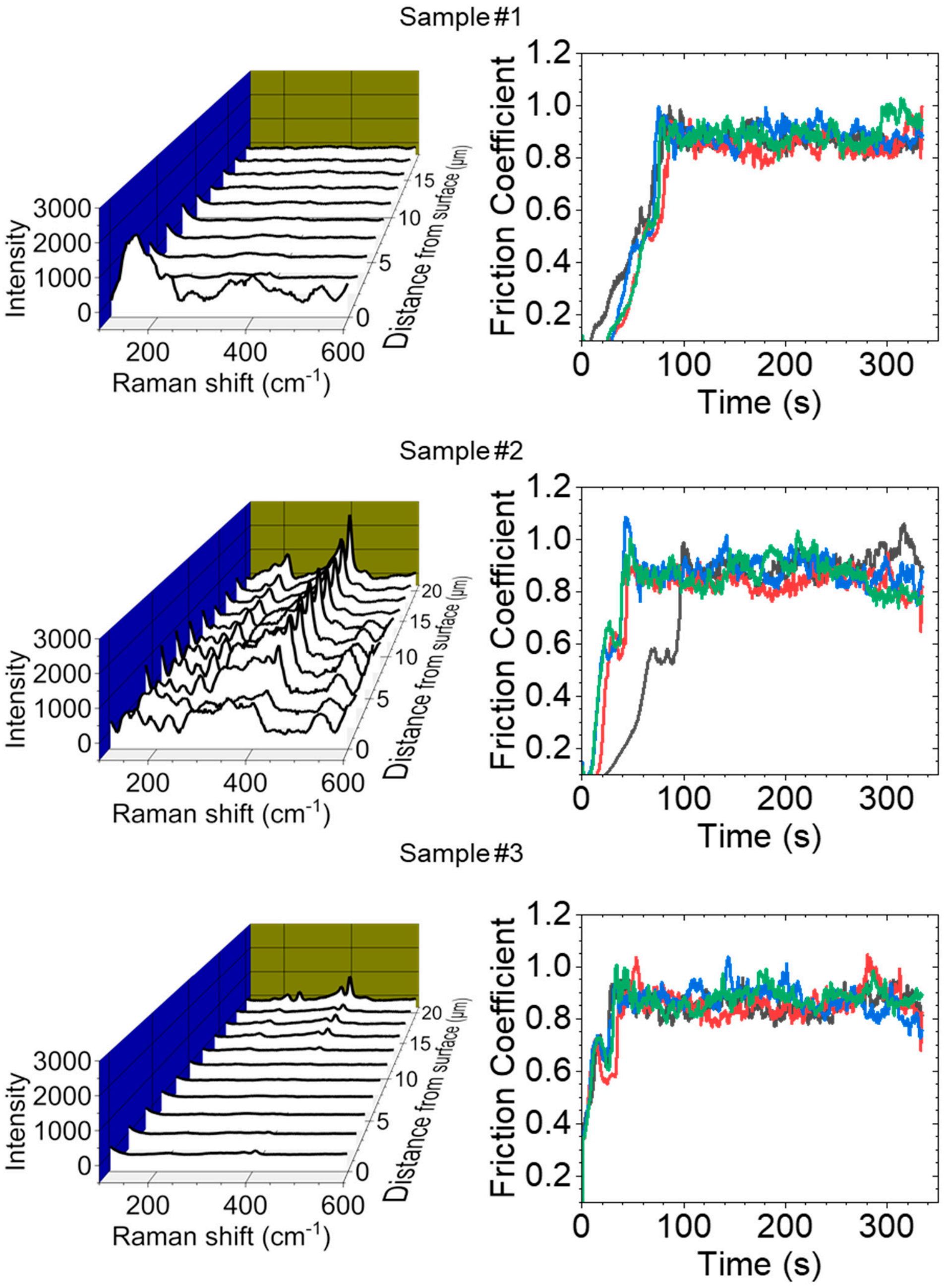

Figure S30. Raman-tribology comparison of 120-minute high-acid anodized Al 6061 samples with HNO_3 pretreatment. Four scratches were performed per sample.

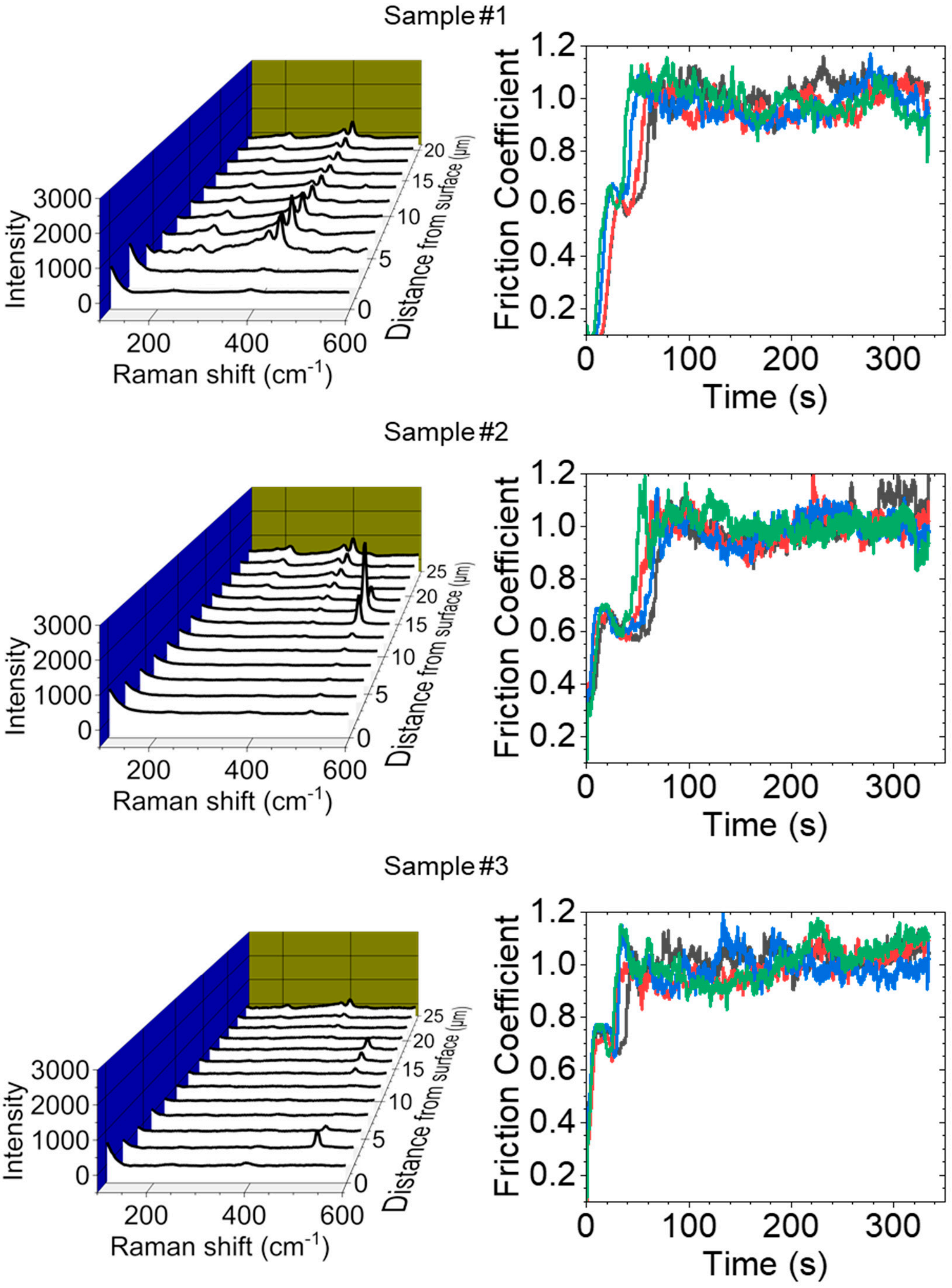

Figure S31. Raman-tribology comparison of 120-minute high-acid anodized Al 1100 samples with no acid pretreatment. Four scratches were performed per sample.

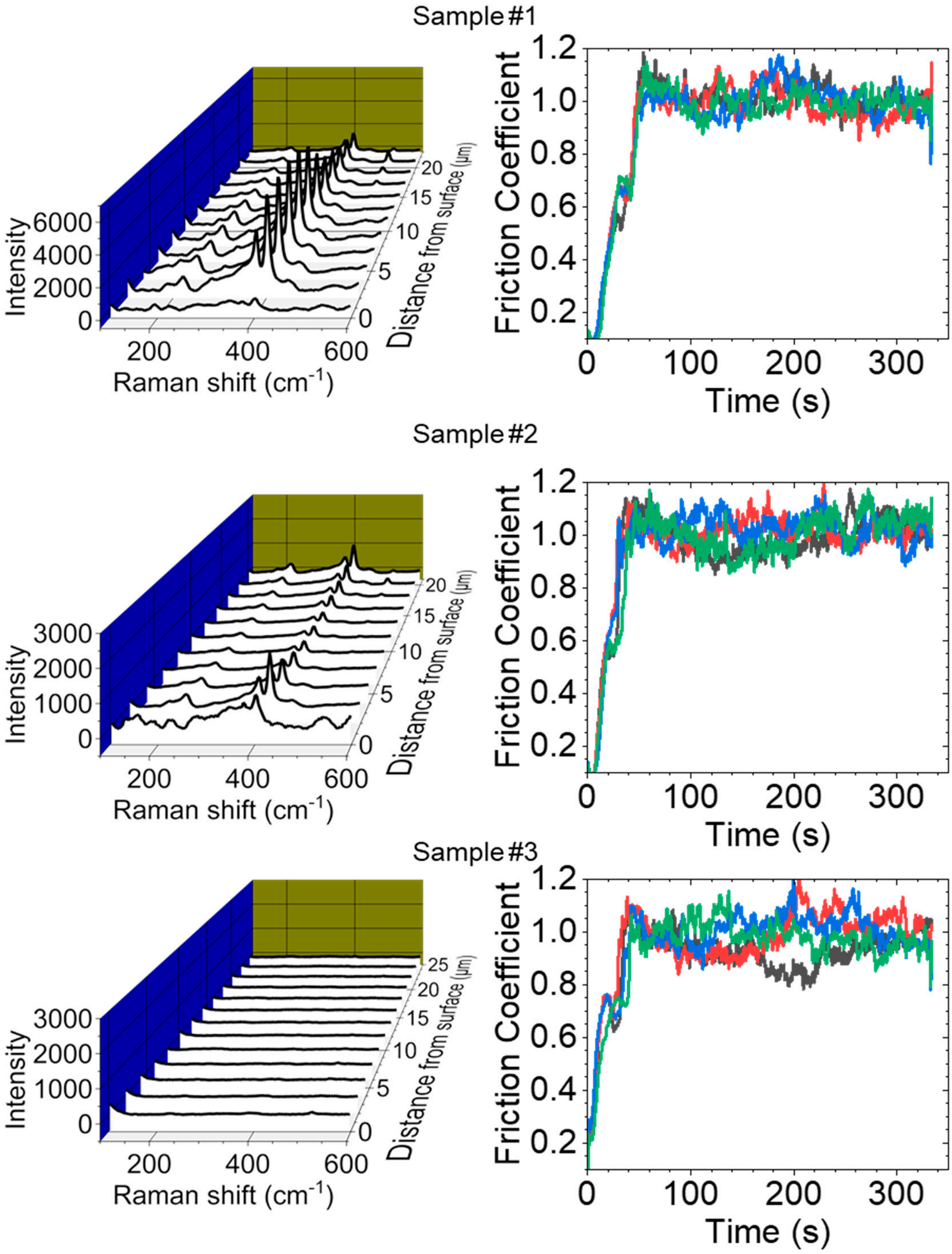

Figure S32. Raman-tribology comparison of 120-minute high-acid anodized Al 1100 samples with HNO_3 pretreatment. Four scratches were performed per sample.
